# Simulations of camera-based single-molecule fluorescence experiments

**DOI:** 10.1371/journal.pone.0195277

**Published:** 2018-04-13

**Authors:** Richard Börner, Danny Kowerko, Mélodie C. A. S. Hadzic, Sebastian L. B. König, Marc Ritter, Roland K. O. Sigel

**Affiliations:** 1 Department of Chemistry, University of Zurich, Zurich, Switzerland; 2 Department of Computer Science, Chemnitz University of Technology, Chemnitz, Germany; 3 Department of Biochemistry, University of Zurich, Zurich, Switzerland; 4 Department of Applied Computer and Biosciences, Mittweida University of Applied Sciences, Mittweida, Germany; University of Toronto, CANADA

## Abstract

Single-molecule microscopy has become a widely used technique in (bio)physics and (bio)chemistry. A popular implementation is single-molecule Förster Resonance Energy Transfer (smFRET), for which total internal reflection fluorescence microscopy is frequently combined with camera-based detection of surface-immobilized molecules. Camera-based smFRET experiments generate large and complex datasets and several methods for video processing and analysis have been reported. As these algorithms often address similar aspects in video analysis, there is a growing need for standardized comparison. Here, we present a Matlab-based software (MASH-FRET) that allows for the simulation of camera-based smFRET videos, yielding standardized data sets suitable for benchmarking video processing algorithms. The software permits to vary parameters that are relevant in cameras-based smFRET, such as video quality, and the properties of the system under study. Experimental noise is modeled taking into account photon statistics and camera noise. Finally, we survey how video test sets should be designed to evaluate currently available data analysis strategies in camera-based sm fluorescence experiments. We complement our study by pre-optimizing and evaluating spot detection algorithms using our simulated video test sets.

## 1 Introduction

Since the initial proof of concept, single-molecule fluorescence techniques, in particular single-molecule Förster resonance energy transfer (smFRET), have proven powerful tools in probing biomolecular structures and dynamics [1–3]. Single fluorescent molecule sensitivity is predominantly achieved using two experimental configurations: (i) confocal microscopy in conjunction with single-photon detection (avalanche photodiodes or photomultiplier tubes) [4,5] and (ii) total internal reflection fluorescence [6] or wide-field microscopy with intensity-based detection using either an electron multiplying charge-coupled device (EM-CCD) [7,8] or a scientific complementary metal-oxide-semiconductor (sCMOS) camera [9]. Camera detection is characterized by a time resolution in the lower millisecond range and a spatial resolution reaching the diffraction limit of visible light [7]. Much higher time resolution can be achieved when photon counting detectors are used in time-correlated single photon counting (TCSPC) experiments with pulsed excitation sources. Conversely, time-binned methods are rather straightforward to implement, comparably inexpensive and, importantly, allow for parallel recording of hundreds to thousands of single molecules at the same time [10,11].Consequently, camera-based fluorescence detection of single-molecules has become a widespread approach for high-throughput acquisition of single-molecule data, especially in the context of smFRET experiments.

Camera-based smFRET generates large and complex data sets that are henceforth referred to as single molecule videos (SMVs). SMVs are characterized by a low net signal and a low signal-to-noise ratio (*SNR*), a high molecule surface density (ρ), short intermolecular distances (*IMD*), and inhomogeneous background profiles. Much effort has been geared towards SMV data analysis in recent years [11–13], but their analysis is at present not standardized. Instead, individual researchers face a host of different data analysis strategies developed by different groups [12,14–17]. Therein, SMV simulations in camera-based SM detection have been reported in the context of kinetic/dwell-time analysis, where method-specific kinetic rates are determined and evaluated against their simulated ground truth, usually as a function of the *SNR *[12,14,18,19]. Although these studies describe the simulation process used, recovering video simulation parameter (VSP) values from the manuscript or supplementary material to reproduce the simulated SMV is typically not straightforward. Independent and reliable assessment of such data analysis strategies requires a common standard to be defined. Thus, the systematic annotation of SMVs with metadata files, would ease and speed up the process of data reproduction. However, standardized, simulated smFRET data test sets annotated with metadata are, to the best of our knowledge, at present not available.

**Fig 1:**
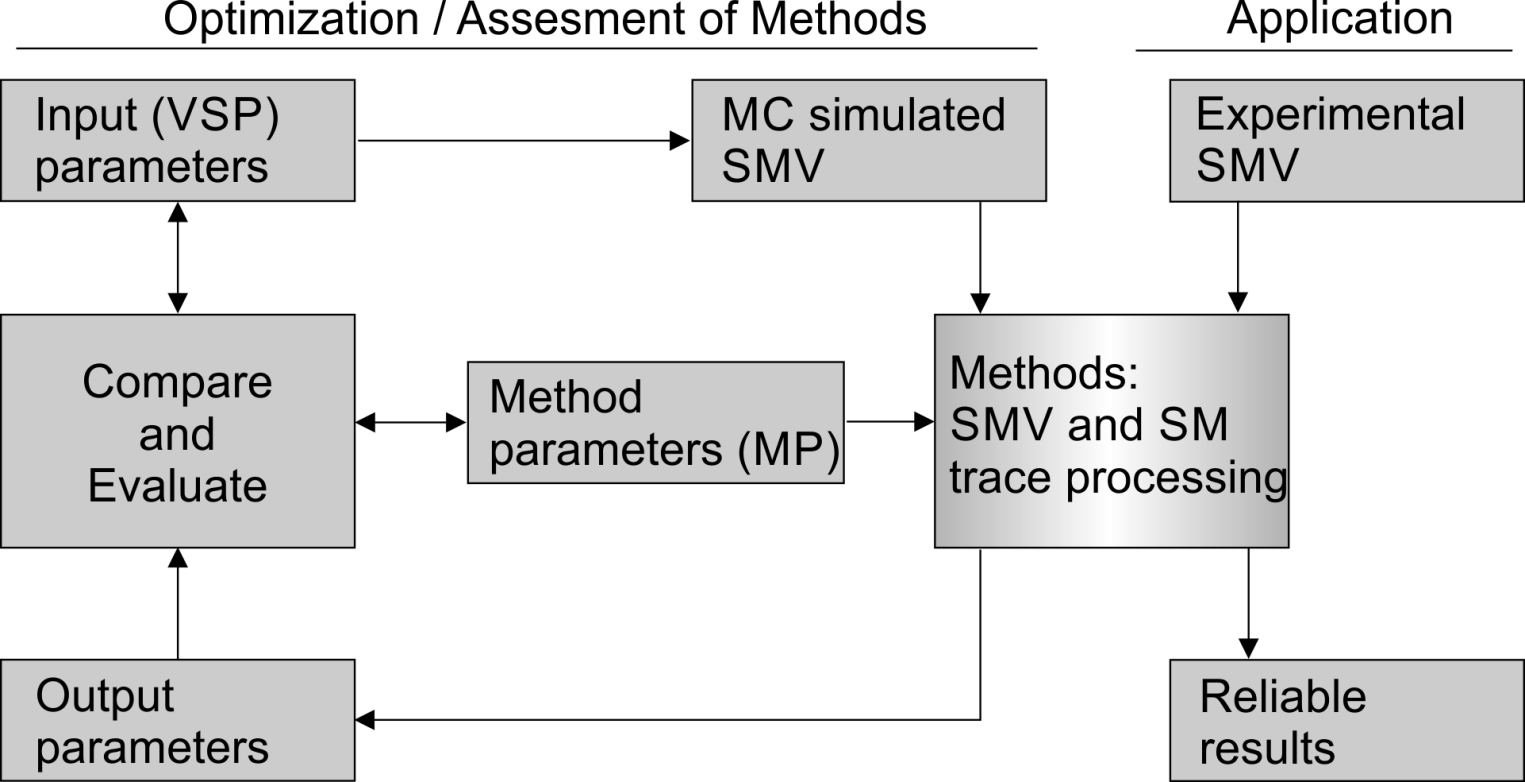
Method parameter optimization and evaluation. Simulated SMVs can be applied to evaluate data analysis algorithms (left) and for comparison with experimental data (right). In the former application, MC simulations are performed to generate a set of SMVs characterized by defined VSPs, followed by analysis using the algorithm to be evaluated. The results (output parameters of the method) are then compared to the input parameters of the simulation to quantify the performance of the algorithm. Method specific parameters (MPs) are varied to maximize the agreement between input and output parameters in order to reach maximum *accuracy* and *efficiency* (see Section 4 for further details). Using pre-optimized parameter sets to analyze experimental data (application) yields reliable results of the molecular system under study.

In the field of computer science, well-annotated and independently designed sets of test data allow the evaluation of individual algorithms [20,21]. Thereby, potential user-bias is minimized, and thus, the evaluation of algorithm via test data sets are accepted as common standard. We believe that standardized, simulated SMV are very suitable as test data sets as they inherently fulfill two conditions: First, they allow the reproducible evaluation of processing algorithms used in smFRET data analysis by providing a set of video simulation parameters (VSP) screening a large experimentally relevant parameter space [18]. Second, simulated SMVs do not contain experimental artifacts due to their unambiguous definition of molecular properties and instrument-specific parameters (**Fig 1**). A comparable approach has been presented by Sage *et al*. for the evaluation of single molecule localization microscopy methods [22] and by Preus *et al*. for the evaluation of background estimators in single molecule microscopy [23].

Here, we provide the theoretical groundwork to generate realistic SMVs covering a large, experimentally relevant VSP space. Specifically, the methodology presented herein addresses (i) the design of thermodynamic and kinetic models, (ii) the distribution of single-molecules in the cameras field of view (FOV) and point-spread function (PSF) modeling using a 2D-Gaussian approximation, (iii) the simulation of photobleaching, (iv) the introduction of signal contributions like background, spectral bleed-through and camera noise and (iv) the SMV export with respect to output file formats and SMV documentation. We present the concepts for performance evaluation of algorithms addressing multiple aspects of SMV data analysis, including SM localization, background correction, intensity time trace generation, trajectory discretization, as well as kinetic and thermodynamic modeling. We use our MATLAB-based SMV simulation tool within our **M**ultifunctional **A**nalysis **S**oftware for **H**andling single molecule **FRET** data (**MASH-FRET**) [13], which is easy-to-use and permits rapid and integrated analysis of camera-based smFRET experiments, to simulate well-annotated SMV test data sets. We apply them to optimize method parameters (MP) of different SM localization algorithms commonly used in the field of camera-based SM fluorescence and compare their performance as a function of the total emitted intensity of single molecules. Both MASH-FRET and the benchmarking data sets are freely available via https://github.com/RNA-FRETools/MASH-FRET.

## 2 The principles of FRET and camera-based smFRET experiments

Förster resonance energy transfer (FRET) denotes the dipole-dipole coupling between a donor fluorophore and an acceptor that is usually, but not necessarily [24,25], also a fluorophore. According to Förster's theory developed in the 1940s, the efficiency of the process, *i*.*e*., the FRET efficiency or transfer efficiency *E*, depends on the inverse sixth power of the interdye distance *r* [26,27]

$$E(r,R_{0})=\frac{1}{1+R_{0}/r^{6}},$$ (1)

where *R*_0_ denotes the Förster radius, *i*.*e*., the distance specific to the FRET pair resulting in a FRET efficiency of 50%. Owing to the pronounced distance dependence of the FRET efficiency, FRET is frequently referred to as a spectroscopic ruler within a range of 3 to 10 nm [28]. For further information on FRET, please refer to dedicated reviews [3,6,15,29].

Camera-based smFRET typically involves total internal reflection (TIR) excitation of surface-tethered biomolecules that are fluorophore labeled [7]. Total internal reflection (TIR) of an incident laser beam can be achieved with a prism or directly through a suitable objective, yielding an evanescent field with an exponentially decaying intensity profile for fluorophore excitation and low out-of-focus fluorescence [6]. Emitted photons are sorted according to their wavelength and detected using a camera. These raw videos are referred to as single-molecule videos (SMVs) throughout this article. In SMVs, fluorophore-labeled biomolecules act as point emitters of light and appear as PSFs in the FOV. In a perfect optical system without spherical aberration, their diffraction pattern can be satisfactorily described by a symmetric 2D Gaussian characterized by the full width at half maximum (FWHM) *w*_det,D/A,0_ [30].

Extracting donor (D) and acceptor (A) emission rates from SMVs permits to approximate the FRET efficiency by the apparent time-dependent transfer efficiency *FRET*(*t*) [7,31]

$$FRET(t)=\frac{I{\left(t\right)}_{D,\;ex}^{A,\;em}}{I{\left(t\right)}_{D,\;ex}^{D,\;em}+I{\left(t\right)}_{D,\;ex}^{A,\;em}},$$ (2)

where $I{\left(t\right)}_{D,\;ex}^{A,\;em}$ and $I{\left(t\right)}_{D,\;ex}^{D,\;em}$ correspond to the photon emission of the acceptor and donor fluorophore at a time point *t*, respectively. In practice, the detected photon emission rates are affected by spectral bleed-through *bt*_D/A_, which results from the detection of donor photons in the acceptor channel, as well as direct acceptor excitation *dE*_A_. Both *bt*_D/A_ and *dE*_A_ can be determined from the overlap of the emission and absorption spectra of the fluorophores taking into account set of optical filters used, although they are generally determined experimentally [7]. Furthermore, the detected photon emission rates contain background signal with different physical and technical origin (see below for further details). Differences in donor and acceptor quantum yields *QY*_D_ and *QY*_A_, as well as detection probabilities of both donor *η*_D_ and acceptor *η*_A_ are accounted for by the correction factor *γ*. A detailed overview on the most relevant corrections and their effects is given in the Supporting Information (**Section C in S1 File**). Applying these corrections eventually yields absolute FRET efficiencies *E*(*r*,*t*) ≡ *FRET*_abs_(*t*), which can be converted into mean inter-dye distances [31–34].

## 3 Simulation of SMVs

**Fig 2:**
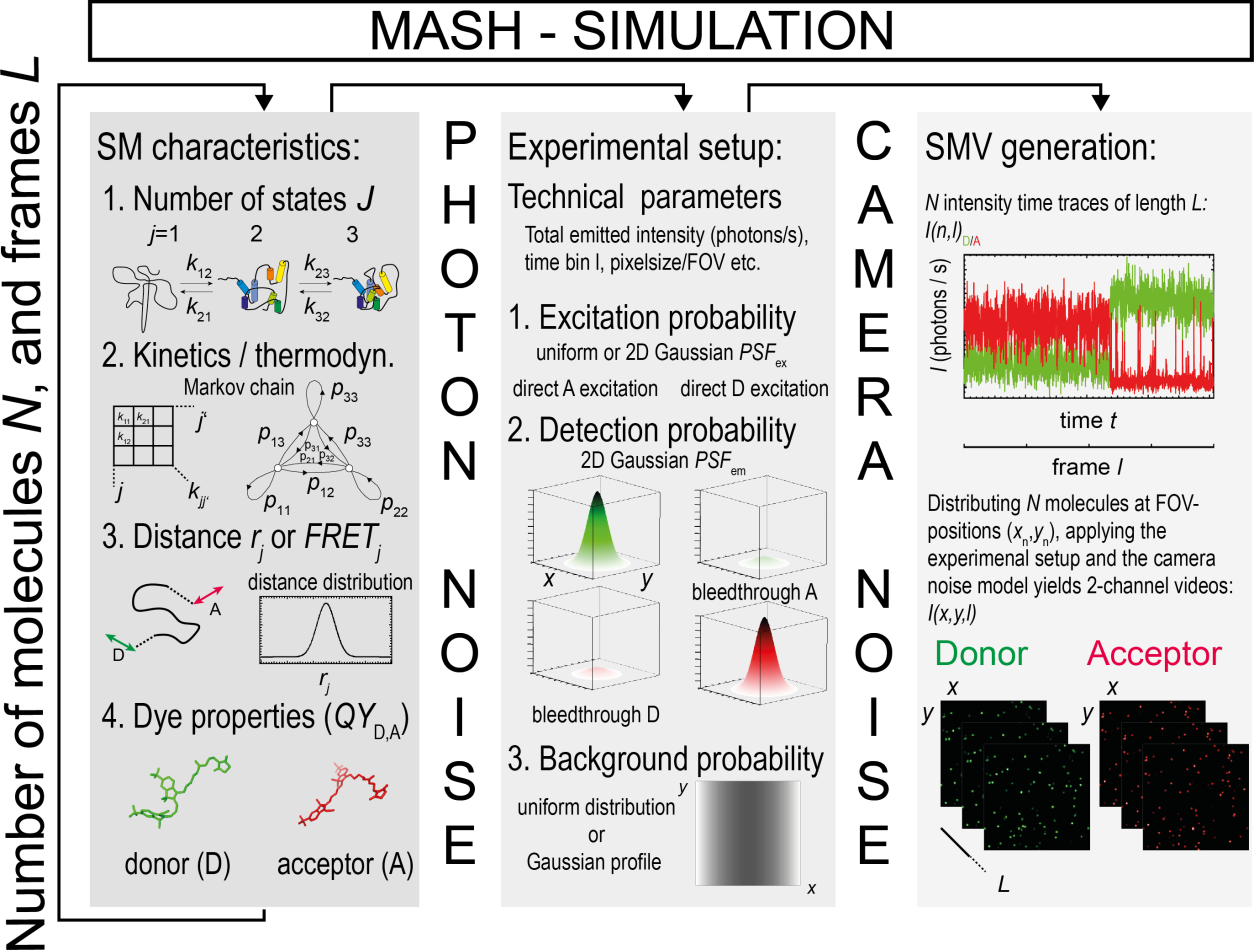
Simulation of SMV. Schematic of smFRET data simulation with TIR illumination and camera detection as implemented in the MASH-FRET simulation tool. A large number of experimental or video simulation parameters can be independently set, ensuring the flexibility of the simulation tool.

FRET efficiencies are calculated from fluorescence intensities. Consequently, we seek to simulate SMVs that contain time-binned fluorescence intensity trajectories, in which the stochastic nature of photon emission is accounted for by a Poisson probability distribution. Simulating SMVs requires the following VSPs to be defined (**Fig 2**): (i) The number of FRET states and the corresponding mean FRET efficiencies adopted by the molecular system under study, (ii) a kinetic model, which describes the transition rates between these FRET states as a Markovian process (**Section 3.2**), (iii) the photophysical parameters of the FRET pair, (iv) the number and location of single molecules within the FOV (**Section 3.3.2**), (v) the PSF model defined by the imaging system (**Section 3.3.3**), (vi) the background signal (**Section 3.3.4**), as well as (vii) a model to describe the noise of the camera (**Section 3.3.6**). The graphical user interface (GUI) of our MATLAB-based SMV simulation tool shown in **Schematic A in S1 File** allows the definition of all the above-mentioned VSPs in a straightforward manner. The simulation tool permits the user to export all video simulation parameters in Matlab-independent exchange file formats.

### 3.1 Workflow to simulate single molecule FRET data

In order to obtain SMVs and FRET efficiency time traces that closely match experimental data, kinetic Monte Carlo (MC) simulations were used to generate *N* fluorescence intensity trajectories (**Fig 2**) [35]. For this purpose, we defined the total number of conformational states (molecular states) *J* as well as the corresponding *J*(*J*-1) monoexponential rate constants *k*_*ij*_ characterizing the interconversion between states *i* and *j*. Each state *j* was assigned to a discrete mean FRET efficiency *FRET*_*j*_. Simulation of each time trace *n* and frame *l* in a time series characterized by a total length *L*, followed by generating SMVs, was achieved in eight steps:

1. Based on the kinetic rates *k*_*ij*_, KMC simulations were performed to determine *FRET*(*n*,*l*) of each molecule *n* for each time bin *l*, *i*.*e*., the observation time a molecule spends in a certain molecular state. In the case of dwell times shorter than the time bin *l*, *FRET*(*n*,*l*) values of multiple states were averaged within the same bin *l*. Kinetic heterogeneity, *i*.*e*., state transitions characterized by multi-exponential kinetics [36], can be simulated by assigning different rate constants *k*_*ij*_ with the same *FRET*_*j*_ value but different states *j*. The kinetic model is further described in **Section 3.2**.
2. Donor and acceptor fluorescence intensities $I{\left(n,l\right)}_{D,\;\mathrm{ex}}^{D,\;\mathrm{em}}$ and $I{\left(n,l\right)}_{D,\;\mathrm{ex}}^{A,\;\mathrm{em}}$ were calculated according to **Eq (2)** based on the discrete *FRET*(*n*,*l*) values and assuming a total emitted donor fluorescence *I*_tot,0_ in the absence of FRET. Even though a constant *I*_tot,0_ for all SMs was used as default setting, it is possible to define molecule-to-molecule variations in *I*(*n*)_tot,0_ to model non-uniform illumination or to create test data sets featuring multiple *SNR* values (**Section 3.3.3**). Further, the probability of donor excitation in absence of FRET was assumed to be independent of time and the relative dipole orientation of both fluorophores. Additionally, the exponential decay in the z-direction of the evanescent wave generated in TIR was neglected, as immobilization typically confines the biomolecules in close proximity to the surface. Differences between quantum yields *QY*_D_ and *QY*_A_, as well as between the detection efficiencies *η*_D_ and *η*_A_ were accounted for by the correction factor *γ* (**Section C in S1 File**). It is noteworthy, that the gamma correction introduces an indirect change of the total emitted fluorescence (**Table C in S1 File**) independent of the user-specified distribution *I*(*n*)_tot,0_ introduced above.
  A frequently observed phenomenon in smFRET are molecule-to-molecule variations. For example, variations can be observed with regard to the mean FRET value of a certain conformational state, the total emitted intensity and quantum yields [3,37]. We modeled cross-sample variability assuming a Gaussian distribution of the underlying VSP values characterized by a defined center and standard deviation *σ*, thus, *FRET*_*j*_ and *σ*_FRET,j_, *I*_tot,0_ and *σ*_Itot,0_, and *γ* and *σ*_γ_. This straightforward approach allows to simulate cross-sample variability in FRET states and trajectory *SNR* originating from instrumental artefacts, but also fluctuations of *QY*, interdye distances, local refractive indices, and fluorophore orientations that lead to *κ*^2^ variations [18,38,39].
3. Instrumental imperfections resulting in direct acceptor excitation and spectral bleed-through were also considered. The respective correction factors are listed in **Section C in S1 File** [40,41].
4. To simulate spontaneous dye photobleaching (see option in **Schematic A in S1 File**), trajectories were truncated using an exponentially distributed photobleaching time.
5. *N* molecules were distributed over a virtual FOV in the respective donor and acceptor channel (**Section 3.3.1**) using either pre-defined or random coordinates (**Section 3.3.2**). The position of each molecule (*x*_n_,*y*_n_) is time-invariant, *i*.*e*., focal drift was assumed to be absent.
6. Pixel values of the respective single molecule coordinates were set to the calculated donor and acceptor fluorescence intensities *I*(*x*_n_,*y*_n_,*l*)_D/A_ in each frame and subsequently convoluted with the PSF of the virtual imaging system as discussed in **Section 3.3.3**. A spatially and/or temporally variable background was added to each frame to account for different sources of background as discussed in **Section 3.3.4** and **Section 3.3.5**. Instrumental imperfections such as focal drift and chromatic aberrations can be simulated too.
7. Photon emission from fluorescent single molecules and background sources was implemented as all-or-none process with a constant probability to emit a photon according to Poisson statistics. In our MC simulations, the resulting photon shot-noise in donor and acceptor emission intensities was accounted for with a Poisson distribution of pixel intensities centered around the expected mean fluorescence intensity (**Section 3.3.6)**.
8. The detected photon signal was finally convoluted with a realistic *SNR* function taking into account the sensitivity, linearity, and temporal noise of camera sensors according to "Emva 1288 Standard Release 3.1" [42] and *Hirsch et al*. [43]. (**Section 3.3.6)**. All simulation parameters were set to default values inspired by values typically observed in an experimental setting (**Table 1**).

**Table 1:** Default values for the simulations carried out with the MASH-FRET simulation tool.

Parameter	Donor (*e*.*g*. Cy3)	Acceptor (*e*.*g*. Cy5)
**Bleed-through**	*bt*_D_ = 7%	*bt*_A_ = 0%
**Direct excitation^a^ [44]**	*dE*_D_ = 0%	*dE*_A_ = 2%
**Total emitted intensity**	*I*_tot,0_ = 100 pc / frame	
**Detection efficiencies^b^**	*η*_D_ = 0.95 (567nm)	*η*_A_ = 0.91 (670nm)
**Quantum yields^c^ [45]**	*QY*_D_ = 0.15	*QY*_A_ = 0.3
**Excitation/ Detection profile**	*w*_ex,x/y,0_ = 60/150 pixel, *w*_det,0_ = 1.5 pixel (2x2 pixel hardware binning)	

^a^ Direct excitation correction can only be performed in ALEX type measurements. We do not simulate ALEX and omit single-labelled species. Thus, the simulation of direct acceptor excitation was performed using the same total emitted intensity of the acceptor as for the donor, *I*_tot,0_.

^b^ The detection efficiencies of cameras are difficult to determine. We take the detection efficiencies of the EMCCD camera Andor iXon3 DU 897D from the manufactures specifications (Oxford Instruments, UK). The overall detection efficiency of the camera was set to the detection efficiency of the donor channel.

^c^ Determined experimentally (*T* = 25°C).

### 3.2 Describing single molecule FRET trajectories as Markov chains

smFRET experiments rely on the assumption that a molecular system capable of adopting *J* conformations yields *J* unambiguously discernable FRET efficiencies. Therefore, we assigned each conformation *j* to a discrete FRET efficiency value *FRET*_*j*_ in our simulations. Here, the thermodynamic equilibrium between these states depends on the kinetic rates *k*_*ij*_ that characterize interconversion between state *i* and *j* for all states *J*. This interconversion was modeled as a Markov chain (**Fig 2**) [46]. In a Markov chain, state transitions are treated as homogenous processes, and the respective *J* × *J* matrix of transition probabilities *p*_*ij*_ to transit from a state *i* to *j* is defined by the transition rates *k*_*i≠j*_ and the frame rate *f* according to [47]:

$$M=\left(\begin{array}{cccc} p_{11} & p_{12} & \cdots & p_{1j}\\ p_{21} & p_{22} & & \\ \vdots & & \ddots & \vdots \\ p_{i1} & & \cdots & p_{ij} \end{array}\right)=\left(\begin{array}{cccc} e^{-\sum_{j=2}^{J}k_{1j}/f} & (1-p_{11})\frac{k_{12}}{\sum_{j=1}^{J}k_{1j}} & \cdots & (1-p_{11})\frac{k_{1j}}{\sum_{j=1}^{J}k_{1j}}\\ (1-p_{22})\frac{k_{21}}{\sum_{j=1}^{J}k_{2j}} & \;e^{-\sum_{j=1\cap j\neq 2}^{J}k_{2j}/f} & & \\ \vdots & & \ddots & \vdots \\ (1-p_{JJ})\frac{k_{i1}}{\sum_{j=1}^{J}k_{ij}} & & \cdots & \;e^{-\sum_{j=1\cap j\neq J}^{J}k_{ij}/f} \end{array}\right).$$ (3)

where *p*_*i = j*_ determines the probability to stay within the same state and *p*_*i≠j*_ to transit from state *i* to state *j*, respectively. For *i* ≠ *j*, rates were assumed to adopt values ≥ 0, whereas rates were set to 0 for *i* = *j*. In the context of this study and for each simulated time trace *n*, we defined the state *i* of time bin *l* = 0 as the state with the highest probability *p*_*i = j*_ according to the probability matrix in **Eq (3)**. We assumed a total length *L* of the observation time and determined the conformational state at each time bin by means of a KMC simulation. Please note that Matlab's built-in function for HMM time series generation (hmmgenerate) parametrized using *p*_*jj’*_ in **Eq (3)** to build a time-resolved kinetic model and to generate the corresponding *FRET* time trace does not allow for time averaged states of single bins or frames, respectively. The simulation of the respective states in the following time bins can be carried out in two different ways: (i) bin wise or (ii) dwell time wise. For (i) we generated a random value from a uniform probability distribution between 0 and 1 and compared it to the cumulative probability vector $P_{i}(j)=\sum_{n=1}^{j}p_{in}$ of the respective state *i* in time bin *l* = 0. Thus, we defined the new state in time bin *l* = 1. The simulation was repeated for each time trace *n* until time bin *L* was reached. For (ii) we set *p*_*i = j*_ = 0, generated a random value and compared it to the probability vector $P_{i}(j)=\sum_{n=1}^{j}p_{in}$. Thus, we obtained the next state *j* and generated the dwell time in state *I* from a monoexponential probability distribution, a hallmark of classical chemical kinetics, characterized by the respective rate coefficient *k*_*ij*_. All time bins within the generated dwell time were set to state *i*. In the case of dwell times exceeding the last time bin, the last dwell time was truncated to the total length *L*. Note that in particular transitions rates *k*_*ij*_ ≥ *f* lead to the detection of artificial states due to time averaging [29]. Therefore, in the case of dwell times shorter than the binning time, we built a time-weighted average of consecutive states in time bin *l* and *l*+1 yielding an averaged FRET value (artificial states) typical for kinetics faster than the exposure time of the camera. Both approaches objectively define the conformational state *j* of the (bio)macromolecule under study *n* for each single time bin *l*. The number of simulation steps in the second approach is drastically reduced, which allows faster simulations. Therefore, it is recommended for the creation of long time traces, traces with small transition rates and traces with large differences between transition rates in order to perform a statistically meaningful kinetic analysis later on.

In intensity-based smFRET experiments, kinetic rates, and thus the transition probabilities, are experimentally accessible from dwell-time analysis [3] or by maximizing the likelihood of a matrix of rate constants in **Eq (3)** according to Gopich and Szabo [48]. Thus, experimentally obtained rate constants can be used to simulate the molecular system under study for comparison, an approach which was presented recently [24].

### 3.3 Instrument specific configuration

In smFRET, the detected signal originates from fluorescent molecules that are only a few nanometers in size. This results in a diffraction-limited spot, whose intensity profile is determined by the characteristics of the optical imaging system, including the microscope objective and beam expander that is typically placed in the detection pathway for magnification (please see **Table C in S1 File** for further examples). In the following, we describe how we accounted for these instrument specific parameters with an appropriate PSF model, by adjusting the number of single molecules in the FOV, the background signal and the noise model of the camera. All of these VSPs can be independently defined in our MATLAB GUI shown in **Schematic A in S1 File**.

#### 3.3.1 Camera and video parameters

SMVs directly dependent on camera-specific parameters, such as the spatial resolution, the pixel size, and the video length, all of which are independent from the actual molecular system. We defined the image size or FOV by the pixel size *d*_pix_ and the dimensions (*x*_res_,*y*_res_) of the camera sensor. Here, the latter depends on the number of horizontal and vertical divisions on the sensor, *x*_res_ and *y*_res_, *i*.*e*., the number of pixel within a Cartesian coordinate system (*x*, *y*). State-of-the-art EMCCD cameras such as an the Andor iXon3 897D (Oxford Instruments, UK) are characterized by a pixel edge width of 16 μm and an *x*_res_×*y*_res_ camera dimension of 512×512 pixel. Note that within an *x*_res_×*y*_res_ image matrix, *x*_res_ defines the number of columns and *y*_res_ the number of rows following Matlab (and other programming languages) notation where an image matrix is defined as lines by rows, *i*.*e*. *y*_res_×*x*_res_. This corresponds to a total sensor area of 8190×8190 μm^2^. In a widefield microscope with 150-fold magnification (objective + beam expander, Table C in S1 File), the total sensor area corresponds to an area of 54×54 μm^2^ in object space. It should be noted that *p* x *p* pixels can be combined to one superpixel (hardware binning), an approach that increases the *SNR* at the expense of decreased spatial resolution. In this study, we performed hardware binning of 2 x 2 pixel and split the detector into two spectroscopic channels characterized by a resolution of 256×128 pixel each. Thus, the simulated object area 54×27 μm^2^ in each spectroscopic channel yields an object size of 0.21×0.21 μm^2^ of the virtual camera pixel in image space perfectly matching the diffraction limit of visible light (Table C in S1 File).

The total number of frames *L* determines the length of the video. The frame rate *f* is a rather arbitrary value in simulated SMV, since greyscale videos are usually saved as *x*_res_ × *y*_res_ × *L* matrices. Here, each *x*_res_ × *y*_res_ image was associated with a binning interval, typically between 10 ms and 100 ms. The binning interval is important for visualizing the SMV with a video player and it affects the outcome of the kinetic analysis (**Section 3.2**).

Photoelectrons generated in response to incident photons are digitized, yielding an integer data type with a given bit rate (*BR*). The *BR* defines the range of values used for the assignment of pixel intensities in units of image counts, electron counts or photon counts. For example, at a *BR* of 14 bit per pixel, the intensity adopts values from 0 to 2^14^−1 = 16383 counts. It should be noted, however, that the *BR* defines a saturated maximum of pixel intensity, which is defined as the maximum of the measured relation between the variance of the gray value and the incident photon flux in units photons/pixel [42]. Thus, the maximum detectable photon flux is given by the variance of the respective image count value of the camera.

We simulated fluorescence intensity trajectories in units of photon counts (pc) per time bin (pc/time bin) or per frame (pc/frame). This enables the evaluation of methods with SMV test data sets independent from the camera specific image count signal. The MASH-FRET analyzing software does not require pc as unit for the intensity; units are arbitrary and can be pc or any other measure for the intensity. However, in the case where pc conversion is not provided by the acquisition software of the camera, *i*.*e*., the detected fluorescence intensity is given in units of image counts (ic) or electron counts (ec) per bin time (ec/time bin = cps) or per frame (ec/frame = cpf), individual measures of the camera should be converted into photon [49]. Equations for converting photon counts into image counts and *vice versa* are provided in **Section 3.3.6** or [42].

#### 3.3.2 Distributing single molecules within the FOV

**Fig 3:**
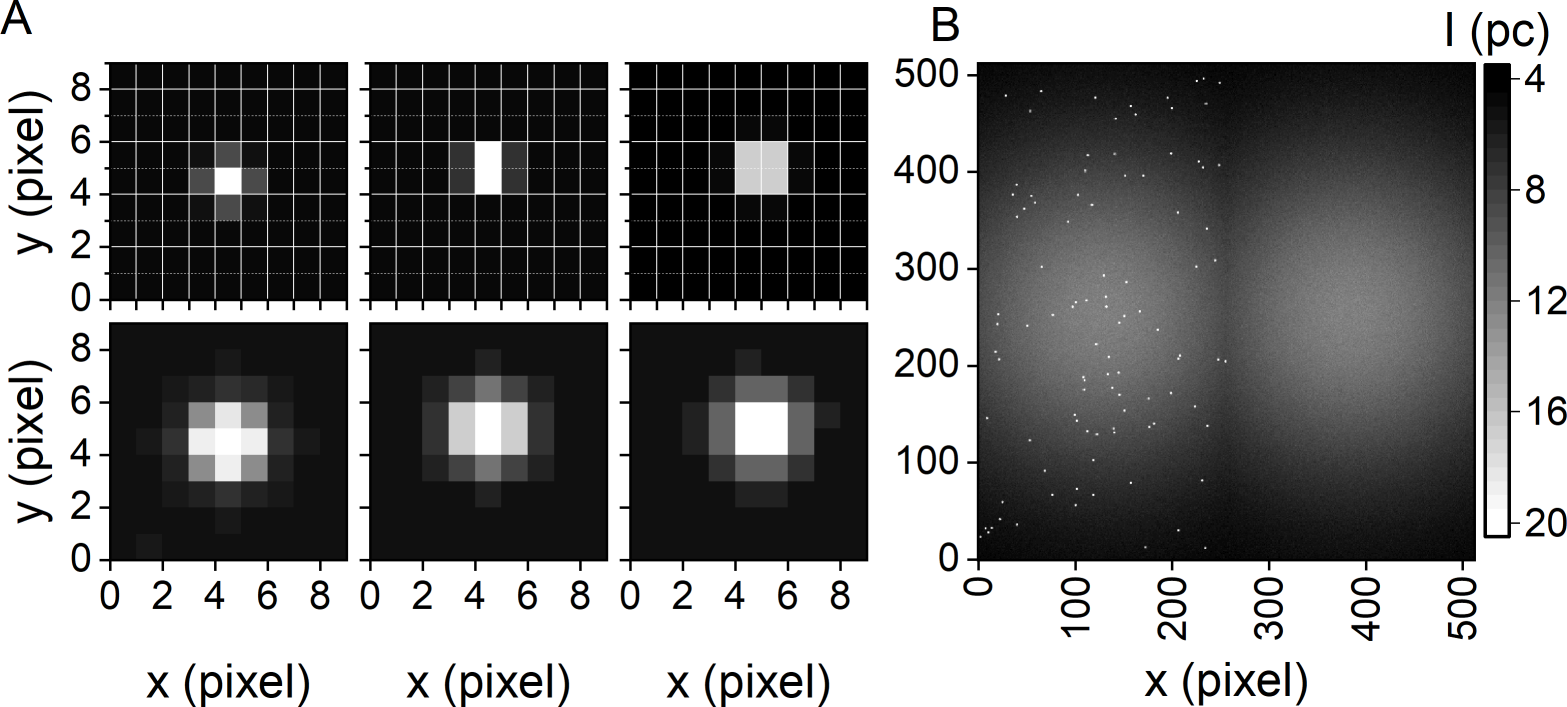
PSF and FOV simulation. (A) Example of simulated PSFs with *w*_det,0_ = 1 pixel (top) and *w*_det,0_ = 2 pixel (bottom) and their appearance depending on their subpixel localization. (B) Representative averaged SMV featuring randomly positioned SMs and an inhomogeneous illumination profile *w*_ex,x,0_ = *w*_ex,y,0_ = 256 pixel.

We used two approaches to distribute *N* simulated molecules within one half of the FOV: (i) We positioned molecules at predefined pixel locations (Fig 3A), yielding regular patterns similar to the ones obtained when nanostructured materials like zero-mode waveguides are used for surface-immobilization [50,51]. Here, overlap between individual PSFs is absent. (ii) We randomly distributed molecules (Fig 3B), typically leading to overlapping PSFs as observed when surface immobilization is achieved **via biotinylated PEG or BSA [36,52].**

To obtain a single molecule density *ρ*, which is defined as the number of SMs per area, and to avoid overlap of different spots, the number of simulated molecules *N* was adjusted as a function of the image dimensions, *i*.*e*., the FOV. To simulate a typical single molecule experiment, we used a low surface molecule density (0.035 μm^-2^ ≤ *ρ* ≤ 0.063 μm^-2^). On average, this corresponds to 82 ≤ *N ≤* 146 molecules distributed over the simulated FOV of 256 x 128 pixel (**Fig 3B)**. Please note that we used subpixel accuracy to distribute molecules within the FOV, resulting in different appearance of single molecules in SMVs depending on their localization relative to the grid of pixels (**Fig 3A)**. Subpixel accuracy in simulated SMVs is particularly important for the evaluation of spot detection algorithms in super resolution microscopy techniques [16,53].

#### 3.3.3 PSF model

The intensity profile of a diffraction-limited spot within the conjugated image plane using an ideal imaging system with a high numerical aperture (*NA* > 1) can be approximated with a normalized, symmetric 3D Gaussian function [30]. Here, we described the detection probability per pixel of a molecule *n* by the detection point spread function *PSF*_det_ in the focal plane:

$$PSF_{\det,\;n,D/A}(x,y,z=0)=\frac{1}{\sqrt{2\pi }w_{0,\det D/A}}\exp\left(-\frac{{\left(x-x_{n}\right)}^{2}+{\left(y-y_{n}\right)}^{2}+{\left(z-z_{0}(t)\right)}^{2}}{2w_{0,\det D/A}{}^{2}}\right),$$ (4)

where the Gaussian width *w*_det,D/A,0_ of the *PSF*_det, *n*, *D*/*A*_ depends on the detection system of the microscope and the emission wavelength of the fluorophores (**Table C in S1 File**). We defined the position of the individual SM, *i*.*e*., the center position (*x*_*n*_,*y*_*n*_), with subpixel resolution, and we implement longitudinal chromatic aberration *z*_0,D/A_ and linear focal drift along the optical axis with *z*_0_(*t*) = *m* ⋅ *t* + *z*_0,D/A_ where *m* is the focal drift rate.

A single fluorophore imaged with a magnifying microscope objective yields, according to Abbe's Law, a diffraction-limited spot of size *w*_det,0_ ≈ *λ*_em_/(2 *NA*). Imaging single Cy3.5 molecules (*λ*_em_ = 600 nm) with a water-immersion objective (60-fold magnification, *NA* = 1.2), results in a diffraction-limited spot size of 15 μm, a value that matches the camera-specific pixel size (**Fig 3A top** and **Table C in S1 File**). Upon increasing the magnification to a factor of 150, a typical value in the context of TIRFM, a diffraction limited spot spreads over a detection area of 3×3 pixel (**Fig 3A bottom**), or in the case of 2x2 pixel hardware binning over 1.5×1.5 pixel. The latter is used as default in our simulation software. It is important to mention that for a microscope-independent evaluation of SM methods, the Gaussian width of the PSF is given in pixel rather than micrometers.

#### 3.3.4 Background simulation

Background signal arises from various environmental and molecular sources, as well as photophysical processes [23]: (i) Environmental light (stray and ambient light) is usually suppressed via blackout-material (blinds, coating, masking tape) along the beam path and especially around the sample and/or the objective. (ii) The incident laser excitation light leads to Rayleigh or Raman scattering from the sample, the optics or even the detectors surface of the CCD camera [7]. The wavelength of scattered excitation light does usually not overlap with the spectral range of interest, *i*.*e*., the emission wavelength of the fluorophores, and can thus be suppressed by a suitable set of optical filters. (iii) Objective and sample chamber autofluorescence depends, for example, on the type and quality of glass. In addition, the sample itself can be autofluorescent when single-molecules are imaged in living cells or cell extracts [54] albeit the contribution of cellular autofluorescence can be reduced by choosing a dye pair that is excited at *λ*_ex_ ≥ 520 nm[55]. Moreover, spurious adsorption of fluorescent impurities to the surface is observed on a regular basis. (iv) Studying intermolecular reactions by smFRET may require the presence of a fluorophore-labeled species at super-picomolar concentrations in solution [36,56]. Thus, freely diffusing and transiently adsorbing fluorescent particles contribute to the fluorescence that adds to the overall background signal. (v) The signal detected by a camera with closed shutter, so called dark images, numerically contributes to the background with a bias-offset*μ*_d_. These dark images are not necessarily homogenous and the particular pattern is camera dependent [43]. Relevant contributions in a particular experiment, however, need to be identified on a case-by-case basis.

In our simulations, we modeled the background as spatially variable *bg*(*x*,*y*)_D/A_, which accounts for background sources (ii-iv). Here, the background is a function of the intensity of the light used for sample excitation. As laser beams with Gaussian cross-section profile are typically used for excitation [10], the excitation profile was described by a normalized, asymmetric 2D Gaussian function *PSF*_ex_, centered in the middle of the respective FOV [10,15]:

$$PSF_{\mathrm{ex}}(x,y,z=0)=\frac{1}{\sqrt{2\pi w_{0,\mathrm{ex},x}w_{0,\mathrm{ex},y}}}\exp\left(-\frac{{\left(x-x_{c}\right)}^{2}}{2w_{0,\mathrm{ex},x}^{2}}+\frac{{\left(y-y_{c}\right)}^{2}}{2w_{0,\mathrm{ex},y}^{2}}+{\left(z-z_{n}(t)\right)}^{2}\right),$$ (5)

where (*x*_c_,*y*_c_) is the center position of the FOV and *w*_0,ex, x/y_ is the width of the Gaussian background profile. Again, the defocusing function *z*_0_(*t*) accounts for lateral chromatic aberration and linear focal drift. It should be noted that this model neglects possible pattern of the dark images (v), as these systematic contribution to the measured image count signal are easily corrected for in an experiment [42,43]. To account for individual experimental conditions (optics, magnification, diameter, etc.) of the incident laser beam, *w*_0,ex,x/y_ can be adapted accordingly depending on the actual TIR profile of the microscope used (**Fig 3B**, related parameters in **Schematic A in S1 File**). To obtain a spatially invariant excitation profile and constant background, *PSF*_ex_ must be set to 1.

#### 3.3.5 Photon flux incident on the camera detector

The total detected intensity *I*(*x*,*y*,*l*)_D/A_ of all incident photons on the camera detector including the contribution of a background signal was defined as

$$I{\left(x,y,l\right)}_{D/A}=I{\left(x_{n},y_{n},l\right)}_{D/A}⊗PSF_{\det,\;D/A}(x,y,z=0)+bg_{D/A}\cdot PSF_{ex}(x,y,z=0).$$ (6)

The intensity per pixel in the respective donor and acceptor channel was calculated by the 2D integral over the corresponding pixel dimensions:

$$I{\left(x,y,l\right)}_{D/A}=\int_{x,y}^{x+d_{pix},y+d_{pix}}I{\left(x',y',l\right)}_{D/A}dx'dy'.$$ (7)

#### 3.3.6 Camera noise model

Camera noise is composed of photon or photoelectron shot noise, amplification noise, spurious charge and camera-specific read-out noise, which contribute in an additive fashion [42,57,58]. Herein, we modeled EMCCD camera noise according to Hirsch *et al*. [43], taking only noise components into account, which are experimentally relevant: (i) We considered shot noise from all light sources contributing to photons reaching the detector pixel *I*(*x*,*y*,*l*)_D/A_. The probability of *n*_ph_ incident photons to be detected on the detector pixel (*x*,*y*) during a frame *l* is given by a Poisson distribution *P*(*n*_ph_;*μ*_ph_ = *I*(*x*,*y*,*l*)_D/A_ characterized by the mean number of incident photons *μ*_ph_. The simulation of sole photon noise is commonly used for the simulation of single photon detection applications [19,45,59–62]. (ii) The second noise contribution is the camera shot noise *σ*_pe_ common to all (EM)CCDs or sCMOS [42,43]. The probability of detecting *n*_ph_ photons, *i*.*e*., producing *n*_pe_ photoelectron, is the quantum efficiency *η* of the detector already included via the gamma correction, and follows a binomial distribution. The joint probability of the Poisson distribution and the binomial distribution is again a Poisson distribution *P*(*n*_pe_;*μ*_pe_ = *ηI*(*x*,*y*,*l*)_D/A_ characterized by the mean number of accumulated photoelectrons. (iii) During the read-out process, electrons are shifted through the pixel of the detector area, the readout-register and the EM register by means of changing electrode voltages. This shift creates unwanted electrons, a so-called clock induced charge (CIC) as one component of spurious charge. The probability of observing *CIC* is very low, though, *CIC* nonetheless adds non-negligible intensity spikes to the detected signal. These may introduce artefacts in SMV analysis. (iv) Thermal noise, as another component of the spurious charge, is usually suppressed by cooling the detector to very low temperatures (-80°C). Therefore, we did not consider it further. According to [43], the number of input electrons *n*_ie_ in the EM register is a convolution of the two Poisson distributions of photoelectrons and spurious charge *P*(*n*_ie_;*μ*_ie_ = *ηI*(*x*,*y*,*l*)_D/A_ + *CIC*) (v) Amplification noise of an EM register with gain *g* was modeled as a gamma distribution *G*(*n*_*oe*_;*μ*_*oe*_ = *gμ*_*ie*_) where the mean number of output electrons *μ*_*oe*_ = *gμ*_ie_ consist of the Poisson distributed sum of all input electrons arriving in the EM register. (vi) Readout-noise *σ*_d_ results from the conversion of the analog electron signal, *i*.*e*., the number of output electrons *n*_oe_, into a discrete image value *μ*_ic_ = *μ*_oe_/*s*. We described readout noise with a Gaussian distribution *N*(*n*_ic_;*μ*_ic_ = *μ*_ic,dark_ + *μ*_oe_/s, *σ*_d_) characterized by a standard deviation *σ*_d_ and the amplifier sensitivity or analog-to-digital factor *s*. We added a constant bias offset *μ*_ic,dark_ which is usually added electronically to avoid negative image counts. (vii) Quantization noise *σ*_q_ of the analog-to-digital converter was neglected, because moderate amplifier sensitivities are typically used. Thus, we yield the Poisson-Gamma-Normal (PGN) noise model of the EMCCD camera described earlier [43]

$$p(n_{\mathrm{ic}};I{\left(x,y,l\right)}_{D/A},\eta_{D/A},CIC,g,s,\mu_{\mathrm{ic},\mathrm{dark}})=\left(P(I{\left(x,y,l\right)}_{D/A},\eta_{D/A},CIC)\circ G(g)\right)*N(\sigma_{d},\mu_{\mathrm{ic},\mathrm{dark}})(sn_{\mathrm{ic}})$$ (8)

**Fig 4:**
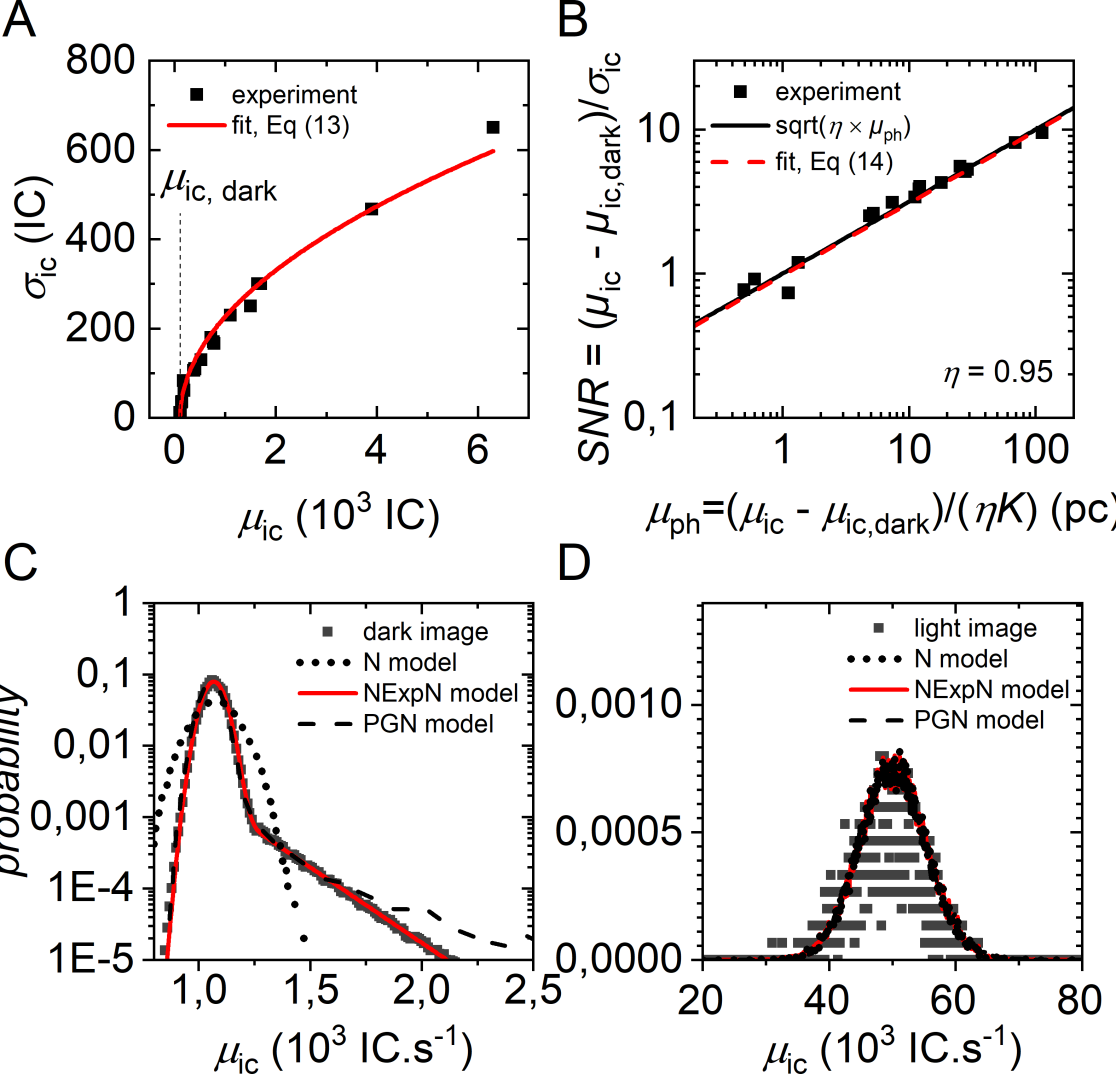
Camera noise simulation. Representative experimental camera noise of an Andor iXon3 DU 897D camera, following EMVA Standard 1288 notation [42]. (A) Standard deviation of camera noise as determined from single pixel temporal intensity fluctuations over 100 frames of single Cy3-labeled RNA with EM gain = 300, *t*_bin_ = 100 ms and a readout rate of 10 MHz. Excitation intensities were varied to yield mean signal intensity rates between 0 and 6000 image counts per frame. Fitting with **Eq (13)** yields: *K* = 57.7 IC e^-1^, *μ*_ic,dark_ = 113 IC e, *σ*_d_ = 0.067 e and *σ*_q_ = 0 IC (B) SNR characteristics of the dark count corrected intensity signal in comparison to an ideal image sensor. The camera units (image counts) were converted into photon counts according to **Eq (12)**. (C) Histogram of the experimentally observed image counts with closed shutter (dark image) for the characterization of CIC noise. Pixel intensities were collected from *L* = 100 video frames (512×512) using the same settings as in (A). Fitting with the NexpN model in **Eq (9)** resulted in μ_oe_ = 1069 IC.s^-1^, *A*_CIC_ = 0.02 and τ_CIC_ = 205 IC s^-1^. PGN noise model parameter (50000 samples): *μ*_ph_ = 0 pc, *CIC* = 0.02 e, others as determined in (A). N noise model parameter (50000 samples): *μ*_ph_ = 0.02 pc (D) Histogram of experimentally observed image counts of a single time trace (1000 frames) of Cy3 labelled RNA. PGN noise model (50000 samples): *μ*_ph_ = 85 pc, *CIC* = 0.02, others as determined in (A). Parameters of the NexpN noise model in **Eq (9)** and the Normal distribution in **Eq (10)** are chosen in the same way as for the PGN noise model.

In difference to reference [43], we found that the CIC noise probability is well described by an exponential distribution *Exp*(CIC) = *A*_CIC_ ⋅ exp(−*I*/*τ*_*CIC*_) that features the terms *A*_CIC_ and τ_CIC_ (**Fig 4**). Here, *A*_CIC_ depends on the EM gain and has usually very small values whereas τ_CIC_ varies with the hardware binning and the vertical clock speed to shift the electrons to the read-out register. Further, we described the number of output electrons *n*_oe_ of the EM register with a normal distribution *N*(*μ*_oe_ = *I*(*x*,*y*,*l*)_*D*/*A*_*η*_*D*/*A*_g, *σ*_oe_) with mean image counts *μ*_oe_ and variance *σ*_oe_^2^ = *μ*_oe._ We approximated the PGN model by a Normal-Exponential-Normal (NExpN) model, where the weighted sum of a Normal distribution and the CIC noise was convoluted with the read-out noise:

$$p(n_{ic};I{\left(x,y,l\right)}_{D/A},\eta_{D/A},A_{\mathrm{CIC}},\tau_{\mathrm{CIC}},g,s,\mu_{\mathrm{ic},\mathrm{dark}})=\left(\begin{array}{l} (1-A_{\mathrm{CIC}})N(I{\left(x,y,l\right)}_{D/A},\eta_{D/A},g)+\ldots \\ \ldots +A_{\mathrm{CIC}}\cdot \exp\left(-I/\tau_{\mathrm{CIC}}\right) \end{array}\right)*N(\sigma_{d},\mu_{\mathrm{ic},\mathrm{dark}})(sn_{\mathrm{ic}})$$ (9)

For sufficiently large EM gains and photon count rates CIC noise becomes negligible. Hence, the NexpN model simplifies to a Normal distribution with mean image counts *μ*_ic_ and variance *σ*_ic_, which we called Normal (N) noise model defined by the parameter set *I*(*x*,*y*,*l*)_*D*/*A*_,*η*_*D*/*A*_,*K* and *μ*_ic,dark_:

$$p(n_{\mathrm{ic}};I{\left(x,y,l\right)}_{D/A},\eta_{D/A},K,\mu_{\mathrm{ic},\mathrm{dark}},\sigma_{\mathrm{ic}})=N(I{\left(x,y,l\right)}_{D/A},\eta_{D/A},K,\mu_{\mathrm{ic},\mathrm{dark}},\sigma_{\mathrm{ic}})(n_{\mathrm{ic}})$$ (10)

In order to estimate the model parameters from experimental values, we recorded the signal of single surface-immobilized, Cy3 labelled RNA molecules with varying fluorescence intensity as described elsewhere [3,36]. The mean signal intensities *μ*_ic_ and standard deviations *σ*_ic_ are shown in **Fig 4A**. Following the nomenclature introduced in reference [43] and the standards for the characterization of image sensors and cameras [42], we defined the temporal variance *σ*_ic_^2^ of the digital signal as the sum of the aforementioned noise sources:

$$\sigma_{\mathrm{ic}}^{2}=\underset{\sigma_{\mathrm{oe}}^{2}}{\underset{︸}{K^{2}\left(\underset{\sigma_{\mathrm{ie}}^{2}}{\underset{︸}{\sigma_{d}^{2}+\sigma_{\mathrm{pe}}^{2}}}\right)}}+\sigma_{q}^{2}$$ (11)

Here, *σ*_d_ combines the noise related to the sensor read-out, the amplifier circuits and the spurious charge that is considered to be independent from the incoming photon signal. *K = g/s* is the overall system gain, *i*.*e*., a linear factor converting the charge into the digital output signal *μ*_ic_ as follows:

$$\mu_{\mathrm{ic}}=K(\mu_{d}+\mu_{\mathrm{pe}})=K(\mu_{d}+\eta \mu_{\mathrm{ph}})=\mu_{\mathrm{ic},\;\mathrm{dark}}+K\eta \mu_{\mathrm{ph}},$$ (12)

where *μ*_d_ denotes the mean number of electrons and *μ*_ic, dark_ = *Kμ*_d_ the mean grey or offset-value of a camera accessed experimentally in the complete absence of light (**Fig 4A and 4C**). The combination of **Eqs (11)** to **(13)** yields the variance of the image count signal of the camera assuming *μ*_pe_ = *σ*^2^_pe_ = *ημ*_ph_:

$$\sigma_{\mathrm{ic}}^{2}=K^{2}\sigma_{d}^{2}+\sigma_{q}^{2}+K\left(\mu_{\mathrm{ic}}-\mu_{\mathrm{ic},\;\mathrm{dark}}\right)=K^{2}\sigma_{d}^{2}+\sigma_{q}^{2}+K^{2}\eta \mu_{\mathrm{ph}}.$$ (13)

The determination of *K* from regression analysis as depicted in **Fig 4A** is known as photon transfer method [57,58]. *K* was further used to calculate the mean number of incident photons *μ*_ph_ as a function of the measured or simulated intensity signal of the camera *μ*_ic_ and *vice versa* in **Fig 4B**. With **Eqs (12)** and **(13)** one can write the *SNR* ratio of the camera signal as follows:

$$SNR=\frac{\mu_{\mathrm{ic}}-\mu_{\mathrm{ic},\;\mathrm{dark}}}{\sigma_{\mathrm{ic}}}=\frac{\eta \mu_{p}}{\sqrt{\sigma_{d}^{2}+\sigma_{q}^{2}/K^{2}+\eta \mu_{p}}}.$$ (14)

**Fig 4B** shows the *SNR* comparison of a real and an ideal sensor. An ideal sensor is characterized by a detection efficiency *η* = 1, negligible dark *σ*_d_ = 0 and discretization noise *σ*_q_/*K* = 0. Here, the *SNR* of the investigated EMCCD as a function of photon counts is close to the square root function of an ideal sensor. We validated the introduced noise models **Eqs (8)** to (**10**) by comparing them to experimental noise distributions in absence and presence of light (**Fig 4 and 4D**). Except for the simple N noise model in **Eq (10)** in case of dark images, we found all noise models to be in good agreement with the experimentally derived signal intensity distribution.

Since recently, low-noise sCMOS cameras featuring megapixel resolution achieve similar or even higher frame rates as their low-resolution CCD counterparts [11]. This allows for a greater SM sensitivity and larger FOVs given the same magnification of the optical system. Even though low-noise sCMOS cameras are devoid of amplification noise, the *SNR* is comparable to EMCCDs. All relevant VSPs related to the camera noise model are implemented in the GUI shown in **Schematic A in S1 File.**

## 4 A generalized approach for comparing algorithms using simulated SMVs

**Fig 5:**
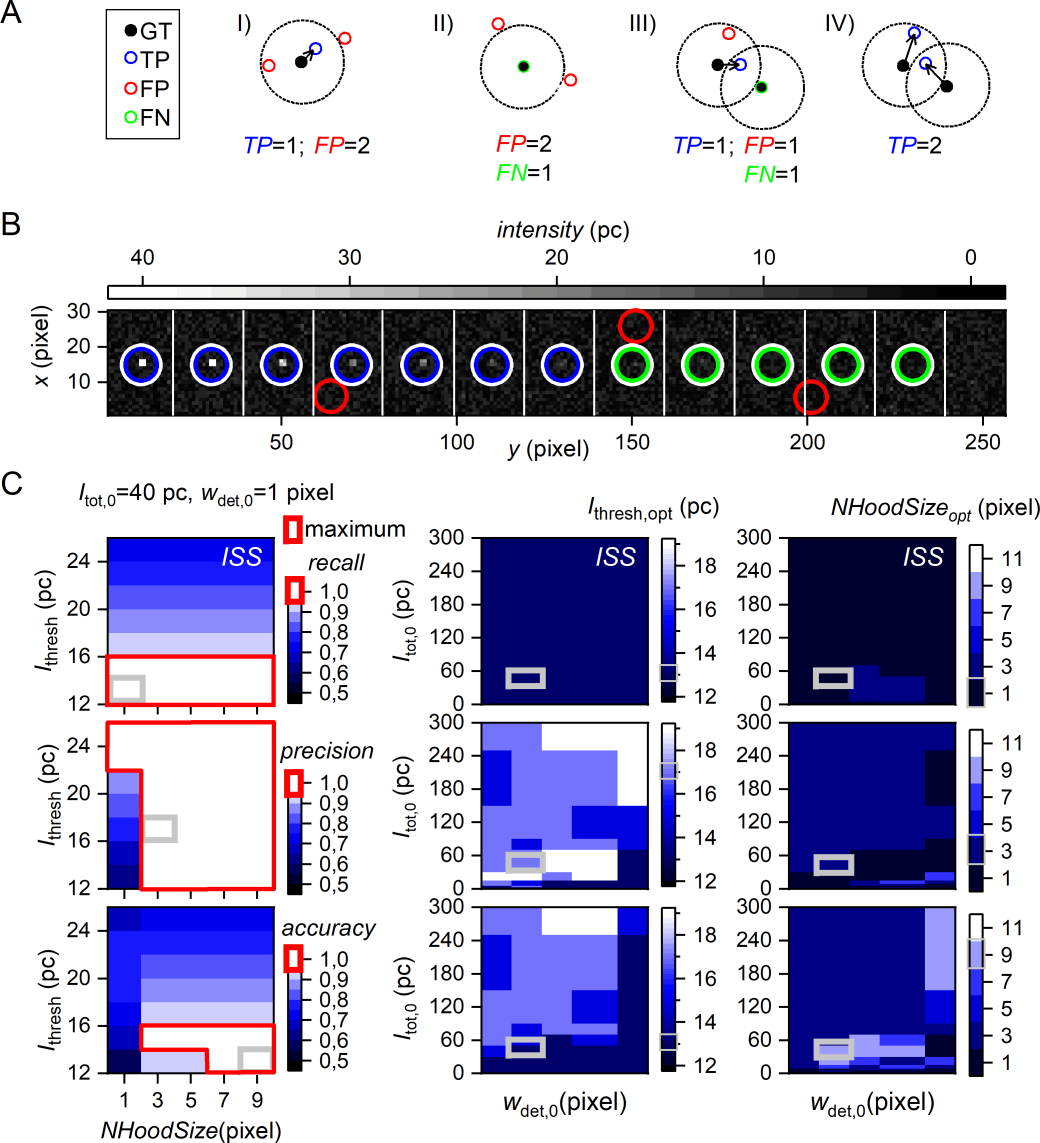
MP Optimization for single molecule spot detection. (A) Classification criteria: Black filled circles mark the ground truth (GT) of simulated SM coordinates (provided as VSP). Dotted circles illustrate a user-defined tolerance radius used for classification of detected SMs (open circles) into I) true positive (*TP*, blue assigned by black arrow), false positive (*FP*, red) and II) false negatives (*FN*, green) based on closest distance to a GT coordinate (greedy approach) and being located within the tolerance radius. Example III) and IV) compare a greedy and Gale-Shapely based approach where both show the same detection of SM but different classifications. For details, see text. (B) A sub-image of 256×30 pixels from a 512×512 pixels video with a total of 24 × 12 single molecules. The total emission intensity (given in photon counts per frame) decreases from the left to the right. White circles mark the *GT* coordinates, blue, red and green mark TP, FP and FN, respectively. (C) Optimization of *ISS* algorithm parameterization for SM detection using SMV category (i) (**Table D in S1 File**) with *w*_det,0_ = 1 pixel. (C, left) Variation of model parameters (MP): 35 combinations of ISS input parameters *I*_thresh_ (intensity threshold) and *NhoodSize* (spot size) and their obtained color-coded *recall*, *precision*, and *accuracy* are shown for molecules of 40 pc total emitted intensity *I*_tot,0_. The respective range for a maximum (= 1) in *recall*, *precision* and *accuracy* are indicated by red bounding boxes. (C, middle and right) Variation of input parameters: Heat maps represent optimal parameterization of *I*_thresh,opt_ (middle) and *NhoodSize*_opt_ (right) to achieve maximum *recall* (1^st^ row), *precision* (2^nd^ row), and *accuracy* (3^rd^ row) as function of PSF size *w*_det,0_ and SM total emitted intensities *I*_tot,0_. Note that *I*_tot,0_ is varied from 1–300 pc within a SMV, while *w*_*det*,*0*_ varies from video to video (category (i), **Table D in S1 File**). The corresponding example parameter optimization results for *I*_tot,0_ = 40 pc and *w*_det,0_ = 1 pixel on the left are highlighted by grey squares in all heatmaps.

Simulated SMVs may serve for comparing, evaluating and/or optimizing algorithms in smFRET data analysis, such as spot detection, background correction, time trace generation, and model selection. Herein, we used simulated SMV as ground truth providing the required information as video simulation parameters to calculate evaluation measures. These measures allow the direct comparison of algorithm performance. Thus, a systematic variation of relevant VSPs enables to find the confidence range of algorithms and their optimal parameterization. We optimized these parameters for a number of commonly used SM localization algorithms (**Fig 5**).

### 4.1 Evaluation measures and classification

To compare different algorithms used in the context of SMV data analysis, we first classified the true values known from the input VSPs as ground truth (GT) (**Fig 5A**, panel I). The output of the algorithm was then classified as true positive (TP, **Fig 5A**, panel II), *i*.*e*., correctly detected values, false positive (FP, **Fig 5A**, panel III), *i*.*e*., erroneously detected non-existing values, false negative (FN, **Fig 5A**, panel IV), *i*.*e*., undetected existing values which are incorrectly classified as non-value, or true negative (TN, omitted in **Fig 5A** as the herein tested algorithms do not classify TNs), *i*.*e*., values correctly classified as non-value. We then quantified the performance of different algorithms by computing the *precision* (also: positive predictive value) and the *recall* (also called sensitivity) [63,64]:

$$recall=\frac{TP}{TP+FN}\;\mathrm{and}\;precision=\frac{TP}{TP+FP}$$ (15)

In addition, the detection efficiency was quantified using the *accuracy*:

$$accuracy=\frac{TP+TN}{TP+TN+FP+FN}$$ (16)

*Recall*, *precision* and *accuracy* adopt values between 0 and 1 and reach 1 when *TP* ≠ *0* and *FP* = *FN* = 0. Since most smFRET-specific algorithms do not produce a *TN* class output, *TN* = 0 in most cases. In the context of method evaluation, the *accuracy* can be used to optimize the input MP in order to assess the method-specific limits of its particular output parameters, to quantify maximum *accuracies* and to test computation times. Since it is generalizable and easily applicable to other algorithms, we believe that it may contribute to the standardization, and hence, the reproducibility of smFRET data analysis.

### 4.2 SMV test sets with different VSPs for method evaluations

We generated a number of SMVs which served as GT for the comparison of different algorithms. We classified the simulated SMVs depending on which VSP was varied and which SMV property was changed, respectively (**Table D in S1 File**). In particular, we varied the following VSPs: (i) The single molecule fluorescence intensity *I*_tot,0_, which affects the *SNR*, (ii) the PSF width *w*_det,0_, which mimics different imaging quantities, (iii) the molecule-to-molecule distances *IMD* and (iv) the molecule surface density *ρ*, both of which affect SM spot overlap, (v) the spatially variable background that affects the signal-to-background ratio, (vi) the trace length *L* and (vii) the ratio of forward/backward transition rate constants *k*_*ij*_ */ k*_*ji*_, both of which affect the kinetic model determination and (vii) the heterogeneity associated with state-specific mean FRET values and the total emitted fluorescence intensity, both of which may influence the inferred model. All simulated SMVs are freely available for download at https://github.com/RNA-FRETools/MASH-FRET for the individual assessment of smFRET-specific algorithms.

### 4.3 Application of simulated SMVs to optimize single molecule detection parameters

To achieve their optimal performance, SM localization algorithms require optimized input MPs (**Fig 1**). We optimized the input MPs of four spot detection algorithms, Houghpeaks (HP), in-series screening (ISS), Schmied (SCH) and TwoTone (2tone) (**Table 2**), and used simulated SMVs of the category (i-ii) suitable for testing the ability of an algorithm to detect SMs given a heterogeneous distribution of the total emitted intensities *I*_tot,0_. All four algorithms apply an intensity cut-off *I*_thresh_ to the SM image prior to SM localization. This ensures that the algorithm can be used at different signal-to-background ratios. All tested detection algorithms process SMVs either sequentially (frame-by-frame) or upon averaging over all frames (frame/time-averaged SMV). The algorithms differ, however, with regard to the number of input parameter, the size parameter related to the PSF width (HP and ISS) or make use of a band pass kernel width (2tone). Different classification algorithms for *TP*, *FP*, and *FN* have been presented: (i) the Greedy approach which classifies *TP*s using the nearest neighbor criterion (type III in **Fig 5A**), and (ii) the Gale-Shapley approach, also known as stable marriage problem, which aims at maximizing *TP*s (type IV in **Fig 5A)** [63,65]. We used the Greedy approach, which is easy to implement and commonly applied. The maximum number of detected spots per frame was fixed to an upper bound larger than the *GT* to allow for the detection of false positives The resulting values for *TP*, *FP*, and *FN* were used to determine the algorithm-specific combination of input MPs yielding maximum *precision*, *recall*, and *accuracy* (**Eqs 15**and **16**).

**Table 2:** Spot detection algorithms tested.

Method	Type/origin	Parameters		Source
**Houghpeaks (HP)**	designed for identifying peaks in images	Intensity threshold *I*_thresh_	11–25 pc (450–800) pc	Matlab image processing toolbox
		Vertical and horizontal spot size *NhoodSize*^a^	1, 3, 5, 7, 9 pixels	
		max. number of spots	9000	
**in-series screening (ISS)**	home-built algorithm inspired from HP	see HP		MASH-FRET
**Schmied (SCH)**	designed for super-resolution microscopy	min. intensity-to-background ratio	1.5–3 a.u. (1.05–3)	[66]
		min. distance to image edge	3	
**TwoTone (2tone)**	designed for analyzing smFRET SMVs	Intensity threshold *I*_thresh_	1–6 a.u.	[15]
		bandpass filter kernel *BpSize*	(4–6) a.u. 1, 3, 5, 7, 9	

Input parameters in brackets are specific to the analysis of time-averaged SMVs.

^a^”The *suppression neighborhood* is the neighborhood around each peak that is set to zero after the peak is identified.” Compare Matlab documentation.

**Fig 5C** illustrates the optimization workflow for the home-written function ISS. Here, the algorithms-specific spot detection parameters *I*_thresh_ and *NhoodSize* were varied (left panel). Decreasing *I*_thresh_ improves *recall* (top row) but deteriorates *precision* (middle row), resulting from a concomitant increases of true and false positives. The maximum values for evaluation measures *recall*, *precision* and *accuracy* cover a range of MP, *I*_thresh_ and *NhoodSize*, depending on the input parameters. These values are highlighted in **Fig 5C** using red bounding boxes for the underlying SMVs of category (i) at *I*_tot,0_ = 40 pc and *w*_det,0_ = 1 pixel. At 40 pc all evaluation measures reach the maximum achievable value of 1, whereas the MP range for the maximum *accuracy* is more limited than for *recall* and *precision*. From this range, maximum *accuracy* is achieved exemplarily for *I*_thresh_ = 13 pc and *NhoodSize* = 9 pixels (**Fig 5C**, solid grey square). *I*_thresh_ is the more critical value for high *recall* values within the examined parameter space in category (ii) simulations co-varying *I*_tot,0_ and *w*_det,0_. The *NhoodSize* parameter of ISS has no impact on the mean *recall*, *precision* and *accuracy for w*_det,0_ *<* 3 pixel and *I*_tot,0_ > 80pcpf. However, for conditions of decreasing SM signal (per pixel), *i*.*e*. towards *w*_det,0_ *=* 3 pixel and decreasing *I*_tot,0_ below 80 pcpf, maximal *precision* and *accuracy* can only be achieved by decreasing *I*_thresh_ and increasing *NhoodSize* (**Fig 5C**, middle and right). Please note that there is a strong drop of the maximum accuracy upon decreasing *I*_thresh_ (**Fig 5C**, left).

In order to optimize the input parameters of the other spot detection algorithms assessed, both *I*_tot,0_ and *w*_det,0_, as well as the horizontal intermolecular distance *IMD* and *w*_det,0_ were co-varied. The results are shown in **Fig B in S1 File**. For a constant fluorescence intensity, the parameter *I*_thresh_ has a greater influence on *accuracy* than *NhoodSize*, in particular if *IMD* >> *w*_det,0._. However, optimum values for *I*_thresh_ and *NhoodSize* depend on the signal intensity *I*(*x*,*y*,*l*), the camera offset *μ*_ic, dark_, and the camera noise *σ*_ic_, and are thus specific to the present set of simulation parameters assessed. The parameter optimization for 2tone is special, because we found two parameter regimes yielding optimal results. The band pass kernel size *BpSize* of 1 and 3 requires *I*_thresh_ parameters of 5.5 and 1.5, respectively. Such an anti-correlated effect between two parameters yielding maximum *accuracy* was demonstrated for ISS at increasing *w*_det,0_ and decreasing *I*_tot,0_ values too. Without going into methodical details, a modest increase of *I*_thresh_ usually reduces *FP*s, which can be a consequence of image artifacts brighter than the local background caused by image processing steps used in a SM detection method. 2tone shows a clear preference of *BpSize* = 1 for the *I*_tot,0_ dependence which is not the case for the *IMD* dependence.

In summary, reducing only the parameter *I*_thresh_ in the four investigated SM localization methods primarily results in more *TP*s and *FP*s, and thus, higher *recall* but lower *precision*. In general, a PSF theoretically leaks out of the ROI defined by the neighborhood size parameter *NhoodSize*, making e.g. ISS and HP prone for the detection of *FP*s at the boundary of the Nhoodsize-ROI. SM localization methods may contain image processing steps (usually implemented as black-box) that entail similar PSF smear effect (e.g. kernel size of 2tone). Thus, highest accuracy is often a compromise between *I*_thesh_ and other parameters like *Nhoodsize*. In smFRET, for low molecule densities where PSF overlap is statistically rare, one might consider prioritizing *precision* over *recall*, a strategy to avoid duplicates (*FP*s located close to *GT*).

**Fig 6:**
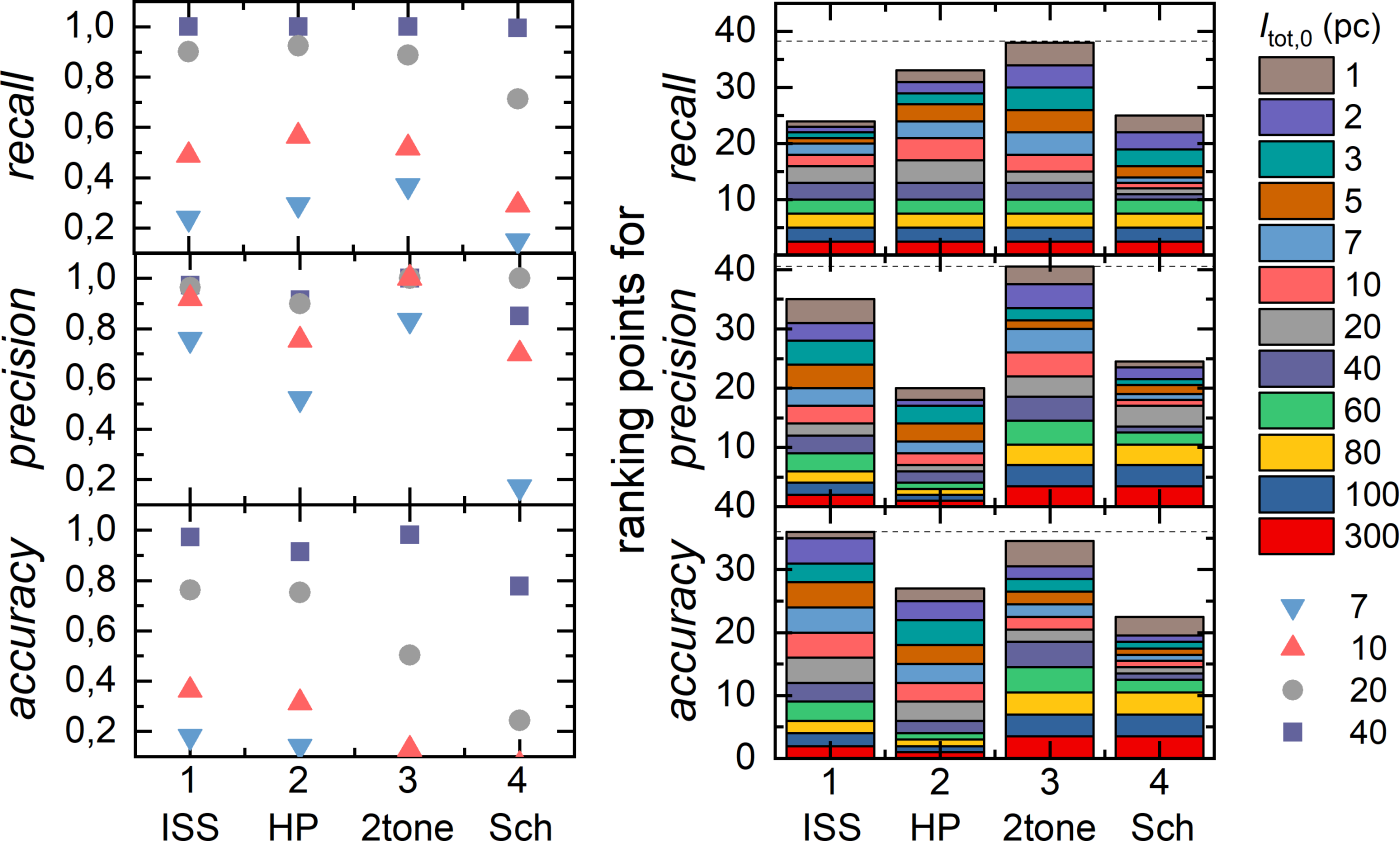
Evaluation of single molecule spot detection after MP optimization. SM detection was performed with four different algorithms, ISS, HP, Sch and 2tone, using simulated SMV of category (i) (**Table D in S1 File**) with and *w*_det,0_ = 1 pixel varying molecules total emitted intensity *I*_tot,0_ form 1 to 300 pc. (left) *Recall*, *precision*, and *accuracy* values of exemplarily chosen *I*_tot,0_ values. (right) Ranking of the four algorithms for *recall*, *precision*, and *accuracy*.

After parameter optimization, SM localization methods are compared in terms of *recall*, *precision* and *accuracy* using their individual set of optimized MP and varying SM total emitted intensity *I*_tot,0_ (**Fig 6A**). To enhance performance contrast, the individual result for *recall*, *precision* and *accuracy* were ranked between the four assessed methods with 1 to 4 points. In case of equal performance, ranking points are equally shared between the respective methods (**Fig 6B)**. Both methods 2tone and ISS have a low false detection rate and thus a high precision. This is is beneficial for the detection of SM spots and a subsequent SM trace generation to avoid “empty” time traces which contain only background signal. ISS achieved best values for accuracy, however, comparable with 2tone. In detail, we find ISS to perform better for higher and 2tone for lower intensity values *I*_tot,0_ (**Fig 6B**). Therefore, ISS and 2tone are both recommended for SM localization in SMVs akin to category (i). Note, that for other categories evaluation results may differ. To provide a deeper insight into the parameter optimization and method comparison presented in this section, we refer to https://github.com/RNA-FRETools/MASH-FRET.

## 5 Conclusion

The first part of this article describes theoretical and practical aspects of simulating camera-based smFRET videos. Simulated SMVs presented herein feature true experimental conditions like realistic camera noise based on Emva Standard 1288 Release 3.1, the simulation of complex kinetic models of multi-state systems potentially showing kinetic heterogeneity, inhomogeneous Gaussian background, and variable SM surface densities. In addition, SM cross sample variability is accounted for in terms of Gaussian distributions of the total emission intensities, FRET states and gamma correction factors. The SMV simulation tool is integrated in our Matlab-based **M**ultifunctional **A**nalysis **S**oftware for **H**andling **s**ingle **m**olecule **FRET** data (**MASH-FRET**), which is freely available for download https://github.com/RNA-FRETools/MASH-FRET.

In the second part, we used the simulation tool to generate well-annotated test data sets that are independent of operating system and software. These test data sets are available online under https://github.com/RNA-FRETools/MASH-FRET and can be used as GT to evaluate computational methods in smFRET data analysis. We illustrated how simulated SMVs can be used to optimize the performance of four spot detection algorithms. For this purpose, we adapted the Greedy approach to categorize detected coordinates as *TP*s, *FP*s, and *FN*s, a classification that was used to define *recall*, *precision* and *accuracy*. This approach provides a standardized way of scoring the performance of spot detection algorithms. We provided a quantitative summary of optimized parameters of the methods assessed as a function of different PSF widths, SM emission intensities and intermolecular distances. We observed that method accuracies and parameterizations of all spot detection algorithms assessed are distinct functions of *w*_det,0_, *I*_tot,0_ and *IMD*. Based on our results we want to emphasize the importance of a careful method parameterization prior to SM detection.

## 6 Software availability

All algorithms have been encoded in a program called **M**ultifunctional **A**nalysis **S**oftware for **H**eterogeneous single molecule **FRET** data **(MASH-FRET**). MASH-FRET was developed with MATLAB 7.12 (R2011a). The compatibility was further tested on Windows 8, 8.1 and 10 with MATLAB 7.12 (R2011a) to 9.2 (R2017a). MASH-FRET is available open source under the GPL 3.0 license for download at https://github.com/RNA-FRETools/MASH-FRET. See the README.md and the wiki on for installation details and software usage.

## Supporting information

S1 File Cumulative supporting information. Included: Abbreviations, Variables and Data Types/Section A; Simulation Software GUI/Section B and Schematic A; FRET theory and correction factors for smFRET type measurements [67,68]/ Section C and Tables A and B; Imaging properties for different optical imaging systems/Section D and Table C; Fitting algorithm and concepts/Section E; Camera noise model–analytical solution/Section F; SMV categories/Section G and Table D; Export including metadata/Section H; Spectral representation of optical elements/Section I and Fig A; Method specific optimization of input parameters/Section I and Fig B.
(PDF)

## References

[1] Ha T, Enderle T, Chemla DS, Weiss S (1996) Dual-molecule spectroscopy: molecular rulers for the study of biological macromolecules. IEEE J. Sel. Top. Quantum Electron. 2 (4): 1115–1128.

[2] Hohlbein J, Gryte K, Heilemann M, Kapanidis AN (2010) Surfing on a new wave of single-molecule fluorescence methods. Phys. Biol. 7 (3): 031001. 20686191 10.1088/1478-3975/7/3/031001

[3] Börner R, Kowerko D, Miserachs HG, Schaffer MF, Sigel RKO (2016) Metal ion induced heterogeneity in RNA folding studied by smFRET. Coord. Chem. Rev. 327–328: 123–142.

[4] Henzler-Wildman KA, Thai V, Lei M, Ott M, Wolf-Watz M, Fenn T et al (2007) Intrinsic motions along an enzymatic reaction trajectory. Nature 450 (7171): 838–844. doi: 10.1038/nature06410 18026086 10.1038/nature06410

[5] Schuler B (2013) Single-molecule FRET of protein structure and dynamics—a primer. J. Nanobiotechnology 11: S2 doi: 10.1186/1477-3155-11-S1-S2 24565277 10.1186/1477-3155-11-S1-S2 PMC4029180

[6] Joo C, Ha T (2012) Single-molecule FRET with total internal reflection microscopy. Cold Spring Harbor Protoc. 2012 (12): 1223–1237. 10.1101/pdb.top072058 23209135

[7] Selvin PR, Ha T (2008) Single-molecule techniques. A laboratory manual Cold Spring Harbor, N.Y.: Cold Spring Harbor Laboratory Press. Vii, 507 p.

[8] Hohlbein J, Craggs TD, Cordes T (2014) Alternating-laser excitation: single-molecule FRET and beyond. Chem. Soc. Rev. 43 (4): 1156–1171. doi: 10.1039/c3cs60233h 24037326 10.1039/c3cs60233h

[9] Saurabh S, Maji S, Bruchez MP (2012) Evaluation of sCMOS cameras for detection and localization of single Cy5 molecules. Opt. Express 20 (7): 7338–7349. 22453414 10.1364/OE.20.007338 PMC3500109

[10] Zhao R, Rueda D (2009) RNA folding dynamics by single-molecule fluorescence resonance energy transfer. Methods 49 (2): 112–117. 10.1016/j.ymeth.2009.04.017 19409995

[11] Juette MF, Terry DS, Wasserman MR, Altman RB, Zhou Z, Zhao H et al (2016) Single-molecule imaging of non-equilibrium molecular ensembles on the millisecond timescale. Nat. Methods 13 (4): 341–344. 26878382 10.1038/nmeth.3769 PMC4814340

[12] Preus S, Noer SL, Hildebrandt LL, Gudnason D, Birkedal V (2015) iSMS: single-molecule FRET microscopy software. Nat. Methods 12 (7): 593–594. 26125588 10.1038/nmeth.3435

[13] Hadzic MCAS, Kowerko D, Börner R, Zelger-Paulus S, Sigel RKO (2016) Detailed analysis of complex single molecule FRET data with the software MASH. Proc. SPIE 9711: 971119.

[14] Greenfeld M, Pavlichin DS, Mabuchi H, Herschlag D (2012) Single Molecule Analysis Research Tool (SMART): an integrated approach for analyzing single molecule data. PloS One 7 (2): e30024 doi: 10.1371/journal.pone.0030024 22363412 10.1371/journal.pone.0030024 PMC3282690

[15] Holden SJ, Uphoff S, Hohlbein J, Yadin D, Le Reste L, Britton OJ et al (2010) Defining the limits of single-molecule FRET resolution in TIRF microscopy. Biophys. J. 99 (9): 3102–3111. doi: 10.1016/j.bpj.2010.09.005 21044609 10.1016/j.bpj.2010.09.005 PMC2965953

[16] Small A, Stahlheber S (2014) Fluorophore localization algorithms for super-resolution microscopy. Nat. Methods 11 (3): 267–279. 24577277 10.1038/nmeth.2844

[17] Blanco M, Walter NG (2010) Analysis of Complex Single-Molecule FRET Time Trajectories In: Walter NG, editor Fluorescence based approaches. Amsterdam [u.a.]: Elsevier, Acad. Press Pp. 153–178. 10.1016/S0076-6879(10)72011-5 PMC3012381 20580964

[18] König SLB, Hadzic MCAS, Fiorini E, Börner R, Kowerko D, Blanckenhorn WU et al (2013) BOBA FRET: bootstrap-based analysis of single-molecule FRET data. PloS One 8 (12): e84157 doi: 10.1371/journal.pone.0084157 24386343 10.1371/journal.pone.0084157 PMC3873958

[19] Chen J, Pyle JR, Sy Piecco, Kurt Waldo, Kolomeisky AB, Landes CF (2016) A Two-Step Method for smFRET Data Analysis. J. Phys. Chem. B. 120 (29): 7128–7132. doi: 10.1021/acs.jpcb.6b05697 27379815 10.1021/acs.jpcb.6b05697

[20] Mykhaylo Andriluka, Leonid Pishchulin, Peter Gehler, Schiele B (2014) 2D Human Pose Estimation: New Benchmark and State of the Art Analysis. IEEE Conference on Computer Vision and Pattern Recognition (CVPR).

[21] Sobral A (2013) BGSLibrary: An open c++ background subtraction library. IX Workshop de Visao Computacional (WVC’2013).

[22] Sage D, Kirshner H, Pengo T, Stuurman N, Min J, Manley S et al (2015) Quantitative evaluation of software packages for single-molecule localization microscopy. Nat. Methods 12 (8): 717–724. doi: 10.1038/nmeth.3442 26076424 10.1038/nmeth.3442

[23] Preus S, Hildebrandt LL, Birkedal V (2016) Optimal Background Estimators in Single-Molecule FRET Microscopy. Biophys. J. 111 (6): 1278–1286. doi: 10.1016/j.bpj.2016.07.047 27653486 10.1016/j.bpj.2016.07.047 PMC5034361

[24] Doose S, Neuweiler H, Sauer M (2009) Fluorescence quenching by photoinduced electron transfer: a reporter for conformational dynamics of macromolecules. ChemPhysChem 10 (9–10): 1389–1398. doi: 10.1002/cphc.200900238 19475638 10.1002/cphc.200900238

[25] Cordes T, Santoso Y, Tomescu AI, Gryte K, Hwang LC, Camará B et al (2010) Sensing DNA opening in transcription using quenchable Förster resonance energy transfer. Biochemistry 49 (43): 9171–9180. doi: 10.1021/bi101184g 20818825 10.1021/bi101184g

[26] Förster T (1948) Zwischenmolekulare Energiewanderung und Fluoreszenz. Ann. Phys. 437 (1–2): 55–75.

[27] Lakowicz JR (2010) Principles of fluorescence spectroscopy. New York: Springer Science+Business Media XXVI, 954 s. p.

[28] Schuler B (2007) Application of Single Molecule Förster Resonance Energy Transfer to Protein Folding In: Bai Y, Nussinov R, editors. Protein folding protocols. Totowa, N.J.: Humana Press Pp. 115–138. 10.1385/1-59745-189-4:115 16957321

[29] Schuler B, Hofmann H (2013) Single-molecule spectroscopy of protein folding dynamics—expanding scope and timescales. Curr. Opin. Struct. Biol. 23 (1): 36–47. 23312353 10.1016/j.sbi.2012.10.008

[30] Zhang B, Zerubia J, Olivo-Marin J (2007) Gaussian approximations of fluorescence microscope point-spread function models. Appl. Opt. 46 (10): 1819. 17356626 10.1364/ao.46.001819

[31] Lee NK, Kapanidis AN, Wang Y, Michalet X, Mukhopadhyay J, Ebright RH et al (2005) Accurate FRET measurements within single diffusing biomolecules using alternating-laser excitation. Biophys. J. 88 (4): 2939–2953. doi: 10.1529/biophysj.104.054114 15653725 10.1529/biophysj.104.054114 PMC1282518

[32] Ha T, Ting AY, Liang J, Caldwell WB, Deniz AA, Chemla DS et al (1999) Single-molecule fluorescence spectroscopy of enzyme conformational dynamics and cleavage mechanism. Proc. Natl. Acad. Sci. U. S. A. 96 (3): 893–898. 9927664 10.1073/pnas.96.3.893 PMC15321

[33] McCann JJ, Choi UB, Zheng L, Weninger K, Bowen ME (2010) Optimizing Methods to Recover Absolute FRET Efficiency from Immobilized Single Molecules. Biophys. J. 99 (3): 961–970. doi: 10.1016/j.bpj.2010.04.063 20682275 10.1016/j.bpj.2010.04.063 PMC2913196

[34] Preus S, Wilhelmsson LM (2012) Advances in quantitative FRET-based methods for studying nucleic acids. ChemBioChem 13 (14): 1990–2001. doi: 10.1002/cbic.201200400 22936620 10.1002/cbic.201200400

[35] Fichthorn KA, Weinberg WH (1991) Theoretical foundations of dynamical Monte Carlo simulations. J. Chem. Phys. 95 (2): 1090.

[36] Kowerko D, König SLB, Skilandat M, Kruschel D, Hadzic MCAS, Cardo L et al (2015) Cation-induced kinetic heterogeneity of the intron-exon recognition in single group II introns. Proc. Natl. Acad. Sci. U. S. A. 112 (11): 3403–3408. 25737541 10.1073/pnas.1322759112 PMC4371910

[37] McCann JJ, Choi UB, Zheng LQ, Weninger K, Bowen ME (2010) Recovering Absolute Fret Efficiency from Single Molecules. Comparing Methods of Gamma Correction. Biophys. J. 98 (3): 186a–187a. 20682275 10.1016/j.bpj.2010.04.063 PMC2913196

[38] McKinney SA, Joo C, Ha T (2006) Analysis of single-molecule FRET trajectories using hidden Markov modeling. Biophys. J. 91 (5): 1941–1951. doi: 10.1529/biophysj.106.082487 16766620 10.1529/biophysj.106.082487 PMC1544307

[39] Iqbal A, Arslan S, Okumus B, Wilson TJ, Giraud G, Norman DG et al (2008) Orientation dependence in fluorescent energy transfer between Cy3 and Cy5 terminally attached to double-stranded nucleic acids. Proc. Natl. Acad. Sci. U. S. A. 105 (32): 11176–11181. doi: 10.1073/pnas.0801707105 18676615 10.1073/pnas.0801707105 PMC2516210

[40] Selvin PR, Ha T (2008) Single-molecule techniques A laboratory manual. Cold Spring Harbor, N.Y.: Cold Spring Harbor Laboratory Press. Vii, 507 p.

[41] Kapanidis AN, Laurence TA, Lee NK, Margeat E, Kong X, Weiss S(2005) Alternating-laser excitation of single molecules. Acc. Chem. Res. 38 (7): 523–533. 16028886 10.1021/ar0401348

[42] Working group, EMVA 1288 Emva Standard 1288 Release 3.1. http://www.emva.org/standards-technology/emva-1288/

[43] Hirsch M, Wareham RJ, Martin-Fernandez ML, Hobson MP, Rolfe DJ (2013) A stochastic model for electron multiplication charge-coupled devices‐from theory to practice. PloS One 8 (1): e53671 doi: 10.1371/journal.pone.0053671 23382848 10.1371/journal.pone.0053671 PMC3561409

[44] Kapanidis AN, Lee NK, Laurence TA, Doose S, Margeat E, Weiss S (2004) Fluorescence-aided molecule sorting: analysis of structure and interactions by alternating-laser excitation of single molecules. Proc. Natl. Acad. Sci. U. S. A. 101 (24): 8936–8941. doi: 10.1073/pnas.0401690101 15175430 10.1073/pnas.0401690101 PMC428450

[45] Steffen FD, Sigel RKO, Börner R (2016) An atomistic view on carbocyanine photophysics in the realm of RNA. Phys. Chem. Chem. Phys. 18 (42): 29045–29055. doi: 10.1039/c6cp04277e 27783069 10.1039/c6cp04277e

[46] Seneta E (1996) Markov and the Birth of Chain Dependence Theory. Int. Stat. Rev. 64 (3): 255.

[47] Borczyskowski C von, Zenkevich EI (2016) Self-assembled Organic-inorganic Nanostructures. Optics and Dynamics: Pan Stanford Pub. 50 p.

[48] Gopich IV, Szabo A (2010) FRET efficiency distributions of multistate single molecules. J. Phys. Chem. B. 114 (46): 15221–15226. doi: 10.1021/jp105359z 21028764 10.1021/jp105359z PMC2988977

[49] Jähne B (2004) Vergleichende Analyse moderner Bildsensoren fur die optische Messtechnik. VDI BERICHTE 1829: 317–324.

[50] Zhu P, Craighead HG (2012) Zero-mode waveguides for single-molecule analysis. Annu. Rev. Biophys. 41 (1): 269–293. 22577821 10.1146/annurev-biophys-050511-102338

[51] Chen J, Dalal RV, Petrov AN, Tsai A, O’Leary SE, Chapin K et al (2014) High-throughput platform for real-time monitoring of biological processes by multicolor single-molecule fluorescence. Proc. Natl. Acad. Sci. U. S. A. 111 (2): 664–669. 24379388 10.1073/pnas.1315735111 PMC3896158

[52] Steiner M, Rueda D, Sigel RKO (2009) Ca2+ induces the formation of two distinct subpopulations of group II intron molecules. Angew. Chem. Int. Ed. 48 (51): 9739–9742. 10.1002/anie.200903809 PMC2864518 19924747

[53] Betzig E, Patterson GH, Sougrat R, Lindwasser OW, Olenych S, Bonifacino JS et al (2006) Imaging Intracellular Fluorescent Proteins at Nanometer Resolution. Science 313 (5793): 1642–1645. doi: 10.1126/science.1127344 16902090 10.1126/science.1127344

[54] König I, Zarrine-Afsar A, Aznauryan M, Soranno A, Wunderlich B, Dingfelder F et al (2015) Single-molecule spectroscopy of protein conformational dynamics in live eukaryotic cells. Nat. Methods 12 (8): 773–779. doi: 10.1038/nmeth.3475 26147918 10.1038/nmeth.3475

[55] Aubin JE (1979) Autofluorescence of viable cultured mammalian cells. J. Histochem. Cytochem. 27 (1): 36–43. 220325 10.1177/27.1.220325

[56] Koh HR, Kidwell MA, Ragunathan K, Doudna JA, Myong S (2013) ATP-independent diffusion of double-stranded RNA binding proteins. Proc. Natl. Acad. Sci. U. S. A. 110 (1): 151–156. 23251028 10.1073/pnas.1212917110 PMC3538233

[57] Janesick J, Klaasen K, Elliott T (1985). In: Dereniak EL, Prettyjohns KN, editors 29th Annual Technical Symposium: SPIE Pp. 7–19.

[58] Janesick JR (2007) Photon Transfer. 1000 20th Street, Bellingham, WA 98227–0010 USA: SPIE.

[59] Börner R, Ehrlich N, Hohlbein J, Hübner CG (2016) Single Molecule 3D Orientation in Time and Space: A 6D Dynamic Study on Fluorescently Labeled Lipid Membranes. J. Fluoresc. 26 (3): 963–975. 26972111 10.1007/s10895-016-1784-5

[60] Nir E, Michalet X, Hamadani KM, Laurence TA, Neuhauser D, Kovchegov Y et al (2006) Shot-noise limited single-molecule FRET histograms: comparison between theory and experiments. J. Phys. Chem. B 110 (44): 22103–22124. doi: 10.1021/jp063483n 17078646 10.1021/jp063483n PMC3085016

[61] Kalinin S, Sisamakis E, Magennis SW, Felekyan S, Seidel CA (2010) On the origin of broadening of single-molecule FRET efficiency distributions beyond shot noise limits. J. Phys. Chem. B 114 (18): 6197–6206. doi: 10.1021/jp100025v 20397670 10.1021/jp100025v

[62] Börner R, Kowerko D, Krause S, Borczyskowski, Hübner CG (2012) Efficient simultaneous fluorescence orientation, spectrum, and lifetime detection for single molecule dynamics. J. Chem. Phys. 137 (16): 164202. 23126703 10.1063/1.4759108

[63] Ovesný M, Křížek P, Borkovec J, Svindrych Z, Hagen GM (2014) ThunderSTORM: a comprehensive ImageJ plug-in for PALM and STORM data analysis and super-resolution imaging. Bioinformatics 30 (16): 2389–2390. doi: 10.1093/bioinformatics/btu202 24771516 10.1093/bioinformatics/btu202 PMC4207427

[64] Tan P, Steinbach M, Kumar V (2005) Introduction to data mining Boston: Pearson Addison Wesley. Xxi, 769 p.

[65] Gale D., Shapley L. S. (1962) College Admissions and the Stability of Marriage. Am. J. Math. Mon. 69 (1): 9–15.

[66] Schmied JJ, Gietl A, Holzmeister P, Forthmann C, Steinhauer C, Dammeyer T et al (2012) Fluorescence and super-resolution standards based on DNA origami. Nat. Methods 9 (12): 1133–1134. 23223165 10.1038/nmeth.2254

[67] Valeur B (2002) Molecular fluorescence Principles and applications. Weinheim, New York: Wiley-VCH. Xiv, 387 p.

[68] Clegg RM (1992) Fluorescence resonance energy transfer and nucleic acids. Methods Enzymol. 211: 353–388. 10.1016/0076-6879(92)11020-j 1406315

