# Supplementary material for: Simulations of camera-based single-molecule fluorescence experiments
Source: PLoS One. 2018 Apr 13;13(4):e0195277. doi: 10.1371/journal.pone.0195277 (PMC5898730; doi:10.1371/journal.pone.0195277)
Supplement: S1 File — Included: Abbreviations, Variables and Data Types/Section A; Simulation Software GUI/Section B and Schematic A; FRET theory and correction factors for smFRET type measurements [67,68]/ Section C and Tables A and B; Imaging properties for different optical imaging systems/Section D and Table C; Fitting algorithm and concepts/Section E; Camera noise model–analytical solution/Section F; SMV categories/Section G and Table D; Export including metadata/Section H; Spectral representation of optical elements/Section I and Fig A; Method specific optimization of input parameters/Section I and Fig B. (PDF) [file pone.0195277.s001.pdf]

# Supporting Information

# Simulations of Camera-Based Single-Molecule Fluorescence Experiments

Short Title: Realistic Simulations of Single Molecule Videos

Richard Börner[1,#,*], Danny Kowerko[2,#], Mélodie C. A. S. Hadzic[1,#], Sebastian L. B. König[1,3], Marc Ritter[4], Roland K. O. Sigel[1,*]

# Equal contributions

* Corresponding authors: richard.boerner@uzh.ch, roland.sigel@chem.uzh.ch

1 Department of Chemistry, University of Zurich, Winterthurerstrasse 190, 8057 Zurich, Switzerland

2 Department of Computer Science, Chemnitz University of Technology, 09107 Chemnitz, Germany

3 Department of Biochemistry, University of Zurich, Winterthurerstrasse 190, 8057 Zurich, Switzerland

4 Department of Applied Computer and Biosciences, Mittweida University of Applied Sciences, Technikumplatz 17, 09648 Mittweida, Germany

# A. Abbreviations, Variables and Data Types

## A.1 Frequently used abbreviations

| abbreviation | meaning |
|---|---|
| A | acceptor |
| ALEX | alternating laser excitation |
| BG | background |
| BP | band-pass |
| CCD | charge-coupled device |
| CIC | clock-induced charge |
| D | donor |
| EM-CCD | electron-multiplier charge-coupled device |
| FN | false negative |
| FP | false positive |
| FOV | field of view |
| FRET | Förster resonance energy transfer |
| FWHM | full width at half maximum |
| GT | ground truth |
| GUI | graphical user interface |
| HMM | hidden Markov model |
| IMD | intermolecular distance |
| KMC | kinetic Monte-Carlo |
| MASH-FRET | multifunctional analysis software for heterogeneous smFRET data |
| MC | Monte-Carlo |
| MD | molecular dynamics |
| pc | photon count |
| pcpf | photon count per frame |
| PSF | point-spread function |
| ROI | region of interest |
| sCMOS | scientific complementary metal-oxide-semiconductor |
| SM/sm | single molecule |

| | |
|---|---|
| **SMV** | single molecule video |
| **SNR** | signal-to-noise ratio |
| **TCSPC** | time-correlated single photon counting |
| **TIR** | total internal reflection |
| **TIRF** | total internal reflection fluorescence |
| **TIRFM** | total internal reflection fluorescence microscope |
| **TN** | true negative |
| **TP** | true positive |
| **VSP** | video simulation parameter |

## A.2 Nomenclature

Variables are formatted in italic.

The variable *ch* indicates the channel “D” or “A” of donor or acceptor, respectively.

The variable *ax* indicates the video or image axis “x” or “y”.

The subscript “0” is used in last position to indicate the simulated value.

The subscript “max” is used  to indicate the maximum value.

The subscript “min” is used to indicate the minimum value.

The subscript “opt” is used to indicate the optimum value.

Average values are indicate in brackets <> or topped by a bar ¯.

| Variable | Description |
|---|---|
| $\sigma$ | standard deviation/variance |
| $\mu$ | mean |
| ***SNR*** | signal-to-noise ratio |
| $\rho$ | single molecule density (in FOV$^{-1}$) |
| ***IMD*** | intermolecular distances (in pixel) |
| $E$ | Förster transfer efficiency |
| $r$ | inter-dye distance (in nm) |

| | |
|---|---|
| $\boldsymbol{R_0}$ | Förster radius (in nm) |
| ***FRET*** | apparent Förster transfer efficiency |
| $\boldsymbol{t}$ | recording time (in s) |
| $\boldsymbol{\gamma}$ | gamma correction factor |
| $\boldsymbol{QY_{ch}}$ | quantum yield of the dye emitting in channel *ch* |
| $\boldsymbol{\eta_{ch}}$ | detection probability of the dye emission in channel *ch* |
| $\boldsymbol{Bt_{ch}}$ | bleed-through coefficient of the donor dye into acceptor channel |
| $\boldsymbol{dE_{ch}}$ | direct excitation coefficient of the acceptor dye upon donor excitation |
| $\boldsymbol{J}$ | number of *FRET* states |
| $\boldsymbol{j}$ | *FRET* state index |
| $\boldsymbol{k_{jj'}}$ | transition probability of a state *j* to a state *j'* |
| $I_{ch,\mathrm{ex}}^{ch',\mathrm{em}}$ | photon emission of the dye emitting in channel *ch'* upon excitation of the dye emitting in channel *ch* (in pc) |
| $\boldsymbol{I_{ch}}$ | fluorescence intensity detected in channel *ch* (in pc/frame) |
| $\boldsymbol{I_{\mathrm{tot}}}$ | total fluorescence intensity (in pc/frame) |
| $\boldsymbol{L}$ | trace length (in No. of frames) |
| $\boldsymbol{l}$ | frame index |
| $\boldsymbol{p}$ | probability |
| $\boldsymbol{P}$ | probability distribution |
| $\boldsymbol{N}$ | number of single molecules in recorded/simulated image |
| $\boldsymbol{n}$ | single molecule index |
| $\boldsymbol{(x, y)}$ | pixel coordinates in recorded/simulated image (in pixel) |
| $\boldsymbol{z_{0,\mathrm{ch}}}$ | lateral chromatic aberration (in pixel) |
| $\boldsymbol{m}$ | focal drift rate (in per frame) |
| $\boldsymbol{w_{\mathrm{det},ch}}$ | point-spread function width (in pixel) |
| $\boldsymbol{w_{\mathrm{ex},ax}}$ | Gaussian width of the inhomogeneous background profile (in pixel) |

| | |
|---|---|
| $\boldsymbol{w_{\mathrm{FRET},j}}$ | Gaussian standard deviation in molecular heterogeneity in $FRET_j$ values |
| $\boldsymbol{w_{\mathrm{tot}}}$ | Gaussian standard deviation in molecular heterogeneity in $I_{\mathrm{tot,0}}$ |
| $\boldsymbol{\kappa^2}$ | dye dipole orientation factor |
| $\boldsymbol{px_{\mathrm{size}}}$ | pixel size (in µm) |
| $bg_{ch,\mathrm{ex}}^{ch',\mathrm{em}}$ | fluorescent background in channel *ch'* upon excitation of the dye emitting in channel *ch*. |
| ***GT*** | number of values belonging to the ground truth |
| ***TP*** | number of true positive detections |
| ***TN*** | number of true negative detections |
| ***FP*** | number of false positive detections |
| ***FN*** | number of false negative detections |
| ***recall*** | recall of detections |
| ***precision*** | precision of detections |
| ***accuracy*** | accuracy of detection |
| $\boldsymbol{N_{\mathrm{pix}}}$ | number of pixels summed for intensity trajectory generation. |

## A.3 Data Types

All calculations are carried out in double precision (floating numbers).

## B. Simulation Software GUI

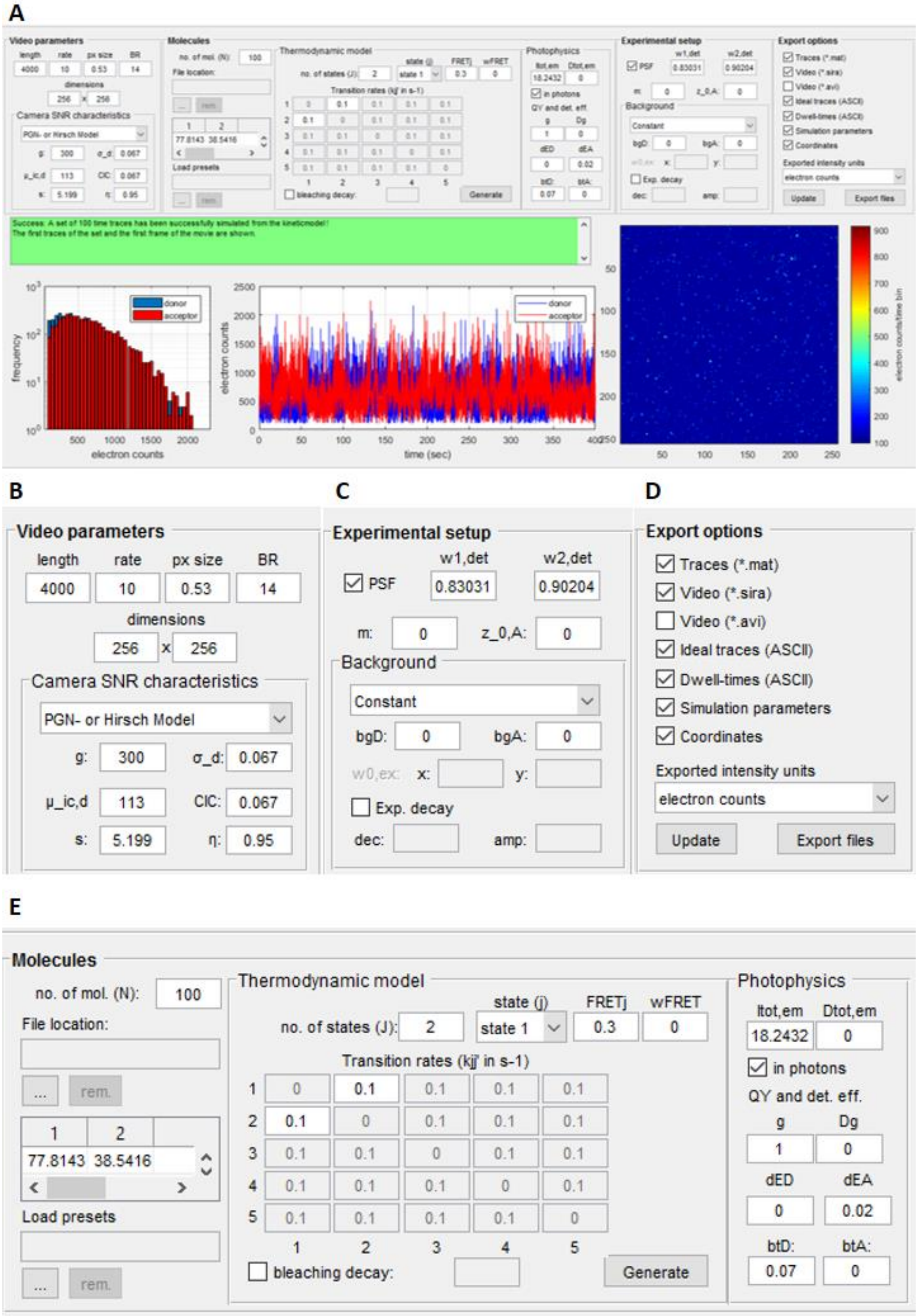


**Schematic A**. Home-written Matlab-based software MASH-FRET: smFRET video simulation tool: (A) graphical user interface and its subsections (B-E). (B) General video parameters and

camera-noise-model characteristics. (C) Experimental setup parameters and background model (D) Export options for different common video export/save formats. (E) Single molecule number and localization, the thermodynamic and kinetic model including FRET state definitions as well as fluorophore characteristics.

## C. Two channel signal separation according to FRET theory

The most fundamental assumption of energy conservation demands the total emitted intensity $I_{\mathrm{tot},0}$ of the donor and acceptor to be constant in time [40]

$$I_{\mathrm{tot},0} = I(t)_{\mathrm{D,ex}}^{\mathrm{D,em}} + I(t)_{\mathrm{D,ex}}^{\mathrm{A,em}} \tag{SI 17}$$

where $I(t)_{\mathrm{D},ex}^{D,em}$ denotes the donor, *e.g.* Cy3 emission (em) upon Cy3 excitation (ex), and $I(t)_{D,ex}^{A,em}$ denotes the acceptor, in particular the transferred energy, *e.g.* Cy5 emission after Cy3 excitation. Assuming Förster type energy transfer or weak coupling for fluorophore distances in the range of 3 – 10 nm [67], the intensity time trace for both, the acceptor and donor signal is given for a certain energy transfer value *FRET* by

$$I(t)_{\mathrm{D,ex}}^{\mathrm{A,em}} = FRET(t) \cdot I_{\mathrm{tot},0} \tag{SI 18}$$

and

$$I(t)_{\mathrm{D,ex}}^{\mathrm{D,em}} = I_{\mathrm{tot},0} - I(t)_{\mathrm{D,ex}}^{\mathrm{A,em}} \tag{SI 19}$$

yielding the time-dependent apparent FRET efficiencies *FRET(t)* as follows:

$$FRET(t) = \frac{I(t)_{D,ex}^{A,em}}{I(t)_{D,ex}^{D,em} + I(t)_{D,ex}^{A,em}}\ . \tag{SI 20}$$

### C.1 Correction factors for smFRET type measurements

The wavelength of the excitation laser source is typically chosen to excite only the donor dye. However, the absorption cross-section of the acceptor is in many cases non-zero, leading to

direct acceptor excitation $dE_A$. In case of alternating laser excitation, the excitation of the acceptor can lead to a direct excitation of the donor. The donor absorption is usually well separated from the acceptor excitation wavelength, leaving the direct donor excitation a rare case.

In practice, the detected photon emission rates are further affected by spectral bleed-through $bt_{D/A}$, which is due to detection of donor fluorescence in the acceptor channel, as well as. Both $bt_{D/A}$ and $dE_A$ can be estimated from the overlay of the emission and absorption spectra of the fluorophores (**Fig A**), though, they are generally determined experimentally [7].

The bleed-through and direct excitation are determined by the experimental setup, in particular by the set of optical components (band pass (bp) and long pass (lp) filter, dichroic mirror (DC) etc.) shaping the fluorophores emission spectrum and the wavelength dependent detection efficiency $\eta$ of the camera (**Fig A**).

The fluorophore quantum yields $QY_D$ and $QY_A$ and the detections efficiency $\eta$ of donor and acceptor emission, which is *e.g.* depending on the detection efficiency of the CCD camera in their respective spectral range, define the gamma correction factor [37]. Within alternating laser excitation (ALEX) [41] the correction factors are accessible in a most convenient way for doubly-labeled constructs using *FRET*-1/*Stoichiometry* density plots [31].

**Table A**. Defining correction factors. Notation for ALEX measurements [40].

| **Correction** | **Donor** | **Acceptor** |
|---|---|---|
| **Background** | $bg_{\mathrm{D,ex}}^{\mathrm{D,em}}$ | $bg_{\mathrm{D,ex}}^{\mathrm{A,em}}$ |
| **Bleed-through** | (Donor only) $bt_{\mathrm{D}} = \frac{I_{\mathrm{D,ex}}^{\mathrm{A,em}}}{I_{\mathrm{D,ex}}^{\mathrm{D,em}}}$ | (Acceptor only) $bt_{\mathrm{A}} = \frac{I_{\mathrm{A,ex}}^{\mathrm{D,em}}}{I_{\mathrm{A,ex}}^{\mathrm{A,em}}}$ <br> $I_{\mathrm{A,ex}}^{\mathrm{D,em}} = 0 \Rightarrow bt_{\mathrm{A}} = 0$ |
| **Direct excitation** | $dE_{\mathrm{D}} = \frac{I_{\mathrm{A,ex}}^{\mathrm{D,em}}}{I_{\mathrm{D,ex}}^{\mathrm{D,em}}}$ <br> $I_{\mathrm{A,ex}}^{\mathrm{D,em}} = 0 \Rightarrow dE_{\mathrm{D}} = 0$ | $dE_{\mathrm{A}} = \frac{I_{\mathrm{D,ex}}^{\mathrm{A,em}}}{I_{\mathrm{A,ex}}^{\mathrm{A,em}}}$ |
| **Gamma correction** | $\gamma = \frac{QY_{\mathrm{A}}\eta_{\mathrm{A,em}}^{\mathrm{A}}}{QY_{\mathrm{D}}\eta_{\mathrm{D,em}}^{\mathrm{D}}}$ | |

With the appropriate correction factors for the background *bg*, the spectral leakage in the donor or acceptor channel for detection *bt*, and the direct excitation *dE*, summarized in **Table A**, we eventually calculate the corrected intensities summarized in **Table B** and yield the definition of the absolute *FRET* value [34]

$$E(t) \equiv FRET(t) = \frac{I(t)''^{\text{A,em}}_{\text{D,ex}}}{I(t)''^{\text{D,em}}_{\text{D,ex}} + \gamma I(t)''^{\text{A,em}}_{\text{D,ex}}} \qquad \text{(SI 21)}$$

which is according to Förster's theory related to the distance *r* between the two fluorophores [26]

$$E(r,t) = \frac{R_0^6}{R_0^6 + r(t)^6} \qquad \text{(SI 22)}$$

The Förster radius $R_0$ is defined by the spectral properties of the fluorophores and is calculated accordingly [68]

$$R_0^6 = \frac{9000(\ln 10)\kappa^2 QY_D}{128\pi^5 N_A n^4} J(\lambda) \qquad \text{(SI 23)}$$

**Table B**. Defining intensity corrections for smFRET measurements.

| Correction | Donor | Acceptor |
|---|---|---|
| | $I^{\text{D,em,0}}_{\text{D,ex}}$ | $I^{\text{A,em,0}}_{\text{D,ex}}$ |
| **Background** | $I^{\text{D,em}}_{\text{D,ex}} = I^{\text{D,em,0}}_{\text{D,ex}} - bg^{\text{D,em}}_{\text{D,ex}}$ | $I^{\text{A,em}}_{\text{D,ex}} = I^{\text{A,em,0}}_{\text{D,ex}} - bg^{\text{A,em}}_{\text{D,ex}}$ |
| **Bleed-through** | $I'^{\text{D,em}}_{\text{D,ex}} = I^{\text{D,em}}_{\text{D,ex}} - bt_{\text{A}} I^{\text{A,em}}_{\text{D,ex}}$ | $I'^{\text{A,em}}_{\text{D,ex}} = I^{\text{A,em}}_{\text{D,ex}} - bt_{\text{D}} I^{\text{D,em}}_{\text{D,ex}}$ |
| | $I'^{\text{D,em}}_{\text{D,ex}} = I^{\text{D,em}}_{\text{D,ex}}$ | |
| **Direct excitation** | $I''^{\text{D,em}}_{\text{D,ex}} = I'^{\text{D,em}}_{\text{D,ex}} - dE_{\text{A}} I^{\text{D,em}}_{\text{A,ex}}$ | $I''^{\text{A,em}}_{\text{D,ex}} = I'^{\text{A,em}}_{\text{D,ex}} - dE_{\text{A}} I^{\text{A,em}}_{\text{A,ex}}$ |
| | $I''^{\text{D,em}}_{\text{D,ex}} = I'^{\text{D,em}}_{\text{D,ex}}$ | |

**Quantum yield & detection efficiency** $I'''^{\mathrm{D,em}}_{\mathrm{D,ex}} = QY_{\mathrm{D}}\eta^{\mathrm{D}}_{\mathrm{D,em}} \cdot I''^{\mathrm{D,em}}_{\mathrm{D,ex}}$ $I'''^{\mathrm{A,em}}_{\mathrm{D,ex}} = QY_{\mathrm{A}}\eta^{\mathrm{A}}_{\mathrm{A,em}} \cdot I''^{\mathrm{A,em}}_{\mathrm{D,ex}}$

## D. Imaging properties for different optical imaging systems.

**Table C**. Overview of typical specifications of standard microscope objectives and standard camera detection parameters for the ideal imaging of diffraction limited spots.

| | **Olympus WI** | **+ 2.5x Beam expander [40]** | **Nikon WI** | **Olympus Oil** | **Nikon Oil** |
|---|---|---|---|---|---|
| **Tube length (mm)** | 180 | / | 200 | 180 | 200 |
| **Magnification (NA)** | 60 (1.2) | 150 | 60 (1.2) | 60 (1.4) | 100 (1.5) |
| **SM object size (nm)**[1] | 250 | 250 | 250 | 214 | 200 |
| **Refractive index** | 1.33 | 1.33 | 1.33 | 1.52 | 1.52 |
| **SM image size (µm$^2$)** | 15x15 | 38x38 | 15x15 | 13x13 | 20x20 |
| $d_{pix}$ [2] **(µm)** | 16 | 16 | 16 | 16 | 16 |
| **Pixel ratio**[3] | 0.9 | 5.6 | 0.9 | 0.7 | 1.6 |
| **with hardware binning**[4] **(2x2)** | 0.2 | 1.4 | 0.2 | 0.2 | 0.4 |

## E. Fitting algorithm and concepts

According to the simulation of FRET data, two standard fitting functions where used throughout the whole manuscript: single-exponential and Gaussian fitting. The fitting was carried out as least square fitting.

## F. Camera noise model – analytical solution

The analytical approximation of the PGN noise model likelihood of an EMCCD is given in [43]. The convolution of the NExpN noise model in Eq. (9) in the main text yields:

---

[1] Theoretical diffraction-limited spot size $\lambda_{\mathrm{em}}/(2NA) \approx w_{0,\mathrm{det\ D/A}}$ (Gaussian approximation) of a SM, *e.g.* Cy3.5 emitting at 600nm.
[2] Pixel size for a iXon3 DU 897 Andor, Oxford Instruments, UK; 8.1x8.1 mm and 512x512 pixel, respectively. Camera port magnification 1x perfect aberration corrected (IX71, Olympus)
[3] Distributed over # pixel. Number of pixels onto the object is imaged.
[4] Distributed over # pixel. Number of pixels onto the object is imaged after hardware binning.

$$p(n_{\mathrm{ic}};\mu_{\mathrm{ic}},\sigma_{ic},A_{CIC},\tau_{CIC})=\begin{matrix}\left(1-A_{CIC}\right)\dfrac{1}{\sqrt{2\pi}\sigma_{ic}}\exp\left(-\dfrac{\left(I-\mu_{\mathrm{ic}}\right)^2}{2\sigma_{ic}^2}\right)+\ldots\\ \ldots+0.5A_{CIC}\exp\left(\dfrac{\sigma_{ic}^2}{2\tau_{CIC}^2}-\dfrac{I-\mu_{\mathrm{ic}}}{\tau_{CIC}}\right)\left(erfc\left(\dfrac{\sigma_{ic}}{\sqrt{2}\tau_{CIC}}-\dfrac{I-\mu_{\mathrm{ic}}}{\sqrt{2}\sigma_{ic}}\right)\right)\end{matrix}\quad. \tag{24}$$

The likelihood of the Gaussian noise model yields:

$$p(n_{\mathrm{ic}};\mu_{\mathrm{ic}},\sigma_{ic})=\frac{1}{\sqrt{2\pi}\sigma_{ic}}\exp\left(-\frac{\left(I-\mu_{\mathrm{ic}}\right)^2}{2\sigma_{ic}^2}\right). \tag{25}$$

The model parameters for both are chosen according to **Section 3.3.6 in the main text**.

## G. SMV categories

- category 1 (variation of total intensity $I_{tot,0}$): Each video contains 24 × 12 SMs, where $I_{tot,0}$ decreases along each row from 300 to 1 pc. Together with the background $I_{tot,0}$ influences the *SNR* of PSF and the intensity time traces. All molecules equally separated by *IMD* = 20 pixels to prevent overlap of adjacent PSFs. These conditions were simulated in 6 videos, for each using a specific $w_{det,0}$ from 0.5 to 3 pixels.
- category 2 (variation of *IMD*): Each video contains pairs of molecules separated by an increasing *IMD* from 0.5 to 20 pixels. $I_{tot,0}$ was fixed at 300 pc and pairs of molecule were spaced in a way to rule out signal overlap between them. These conditions were simulated in 6 videos, for each using specific $w_{det,0}$ values from 0.5 to 3 pixels.
- category 3 (variation of molecule surface density $\rho$): 5 conditions were simulated with increasing number $N$ of molecules from 100 to 3000 molecules/FOV. Molecular coordinates were distributed randomly over the FOV with a constant $I_{tot,0}$ of 100 pc/frame. For each of these 5 molecule density conditions, 6 PSF widths $w_{det,0}$ covering the range from 0.5 to 3 pixels were simulated, giving 30 SMVs in total.

- category 4 (variation of background): Gaussian background profiles were changed varying $w_{0,ex,\ x/y}$ from 128 to 1024 pixels to assess the robustness of background correction methods against inhomogeneous background profiles. $FRET = 0$ was chosen to calculate background values also from the acceptor channel which has the same background profile but shifted horizontally by +256 pixels.
- category 5 (variation of the *SNR*): the SNR is increased from 1 to 7 by rising $I_{tot,0}$, in order to evaluate its impact on the accuracy of state determination methods.
- category 6 (variation of the no. of frames $L$, the closing rate $k_{fast,0}$, the *SNR* via the total fluorescence intensity $I_{tot,0}$, the distribution width $w_{FRET}$ in state-specific *FRET* values and the distribution width $w_{tot,0}$ in molecule-specific $I_{tot,0}$): the five data qualities are separately varied to disentangle their effect on the step detection accuracy, giving five SMV sets for a total of 27 SMVs. The heterogeneity in state-specific *FRET* values is varied by changing the width $w_{FRET}$ of the Gaussian distribution they are generated from. A similar approach is used for the molecule-specific total fluorescence $I_{tot,0}$, varying the width $w_{tot,0}$.
- category 7 (variation of the number of states $J$): computation time was measured in the case of state determination and step detection, for eight SMVs respectively characterized by a number of states between 2 and 9. The FRET states were evenly distributed between 0 and 1.

**Table D**. VSPs categorized for different methods. Red: VSPs to vary. In cases where a parameter is evaluated, its standard default value is highlighted in bold.

| VSP \ SMV category | category 1 | category 2 | category 3 | category 4 | category 5 | category 6 | category 7 |
|---|---|---|---|---|---|---|---|
| **No. of movies** | 5 | 6 | 30 | 4 | 6 | 27 | 5 |
| **Varied parameter** | $I_{tot,0}$, $w_{det,0}$ | $IMD$, $w_{det,0}$ | $\rho$, $w_{det,0}$ | $bg$(x,y) | $I_{tot,0}(SNR)$ | $I_{tot,0}(SNR)$, trace length $L$, $k_{fast,0}$, $w_{FRET,j}$, $w_{tot,0}$ | - no. of states $J_0$ |
| **FOV** | 512×512 | 512×120 | 512×512 | 512×512 | 256×256 | 256×256 | 256×256 |
| **No. of states *J*** | single state | single state | single state | single state | 4 | 2 | 2 |
| **Camera noise** | Poisson | Poisson | Poisson | Poisson | NExpN model $A_{CIC}$ = 0.03, $\tau_{CIC}$ = 0.98 pc | NExpN model $A_{CIC}$ = 0.03, $\tau_{CIC}$ = 0.98 pc | NExpN model $A_{CIC}$ = 0.03, $\tau_{CIC}$ = 0.98 pc |
| **hom. *bg* (pc)** | 4 | 4 | 4 | 8 | 0 | 0 | |
| **inhom. *bg* (pc)** | no | no | no | 128,256,512, 1024 | no | no | no |
| **$w_{ex,x/y,0}$ (pixels)** | | | | | | | |
| **No of frames *L*** | 100 | 1000 | 1000 | 1000 | 4200 | **4200** 70, 140, 350, 700, 7000, 16000, 64000 | 4200 |
| **No. of molecules *N*** (distributed randomly or on rows × cols) | 288 (24 × 12) | 48 (8 × 6 ) | 100, 300, 600, 900, 3000 (rand.) | 100 (rand.) | 100 | 100 | 100 |
| **$I_{tot,0}$ (pc/frame)** | 100 | 1-300 | 100 | 100 | **34.6** 3, 7, 16, 28, 48, 52, 97 | **34.6** 3, 7, 16, 28, 48, 52, 97 | 34.6 |
| **$w_{tot,0}$ (pc/frame)** | 0 | 0 | 0 | 0 | 0 | **0** 1.7, 3.5 6.9 70.4 17.3 | 0 |
| **$w_{det,x/y,0}$ (pixels)** | 0.5, 1, 1.5, 2, 3 | 0.5, 0.75, 1, 1.5, 2, 3 | 0.5, 0.75, 1, 1.5, 2, 3 | 1.5 | no | no | no |
| **photo-bleaching (frame)** | no | no | 100 | 100 | no | no | no |
| **$FRET_{j,0}$** | - | - | - | - | 0.8, 0.6, 0.45, 0.3 | 0.3, 0.7 | evenly [0,1] |
| **$w_{FRET,j}$** | 0 | 0 | 0 | 0 | 0 | **0** 0.01, 0.025, 0.05, 0.075, 0.1, 0.15 | 0 |
| **$k_{jj}$ (frame$^{-1}$)** | - | - | - | - | $k_{12}$ = 0.05, $k_{12}^{-1}$ = 0.05 $k_{23}$ = 0.13, $k_{23}^{-1}$ = 0.13 $k_{34}$ = 0.03, $k_{34}^{-1}$ = 0.03 s$^{-1}$ | **$k_{slow,0}$ = 0.05** ($k_{fast,0}$ = 0.02, 0.04, 0.1, 0.2, 0.4, 1, 2, 10, 20) **$k_{slow,0}$ = 0.02** | $k_{jj'}$ = 0.05 $k_{jj'}^{-1}$ = 0.02 |
| | | | | | | | 5.2 |

## H. Export including metadata

The key export options of our SMV simulation tool are illustrated in **Schematic A.A**. SMVs can be exported as Matlab files or as comma-separated structures (*.csv) independent of the programming language. All VSPs are stored in a log-file to preserve the metadata of our simulation. Furthermore, SMVs can be saved as uncompressed avi files in 8 or 16 bit or using the MASH-FRET-internal format (*.sira) which contains all metadata information. Additionally, SM coordinates can be exported to evaluate SM localization methods. SM kinetics can be exported as dwell-time files including the dwell durations, the corresponding FRET states, the noise-free intensity time traces and the total emitted intensity $I_{tot,0}$. Please note that we refrain from exporting the shot-noise and camera noise broadened intensity time traces, as they are obtained after movie processing and depend on the number of pixel summation.

## I. Figures

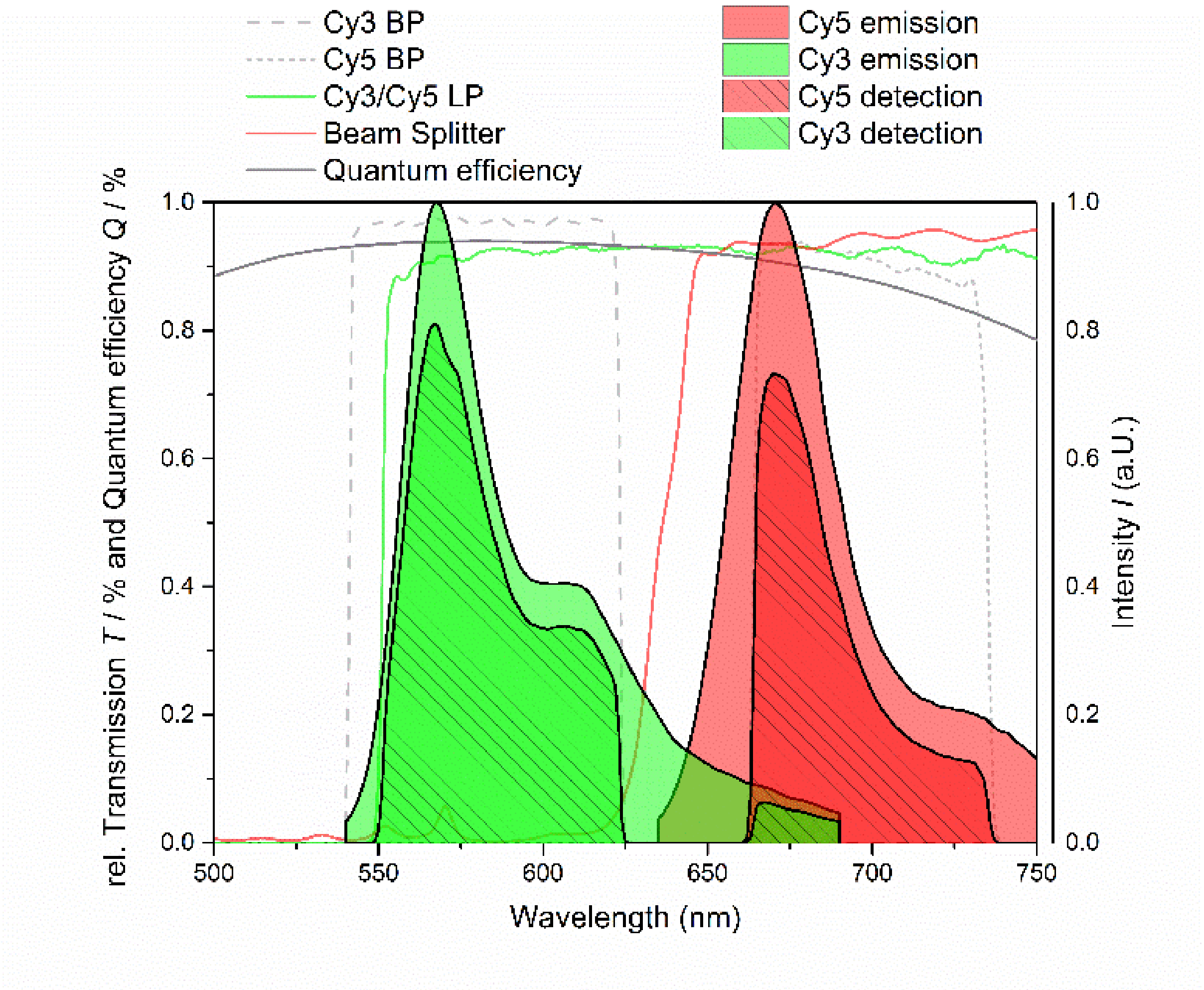


**Fig A**. Spectral representation of all optical elements enclosed in the detection pathway as well as the detection efficiency of a standard EMCCD camera shaping the fluorescence emission spectrum of both FRET labels, the donor *e.g.* Cy3 and the acceptor *e.g.* Cy5. Graphical visualization of the bleed-through and cross talk by the normalized integral in the individual color channels.

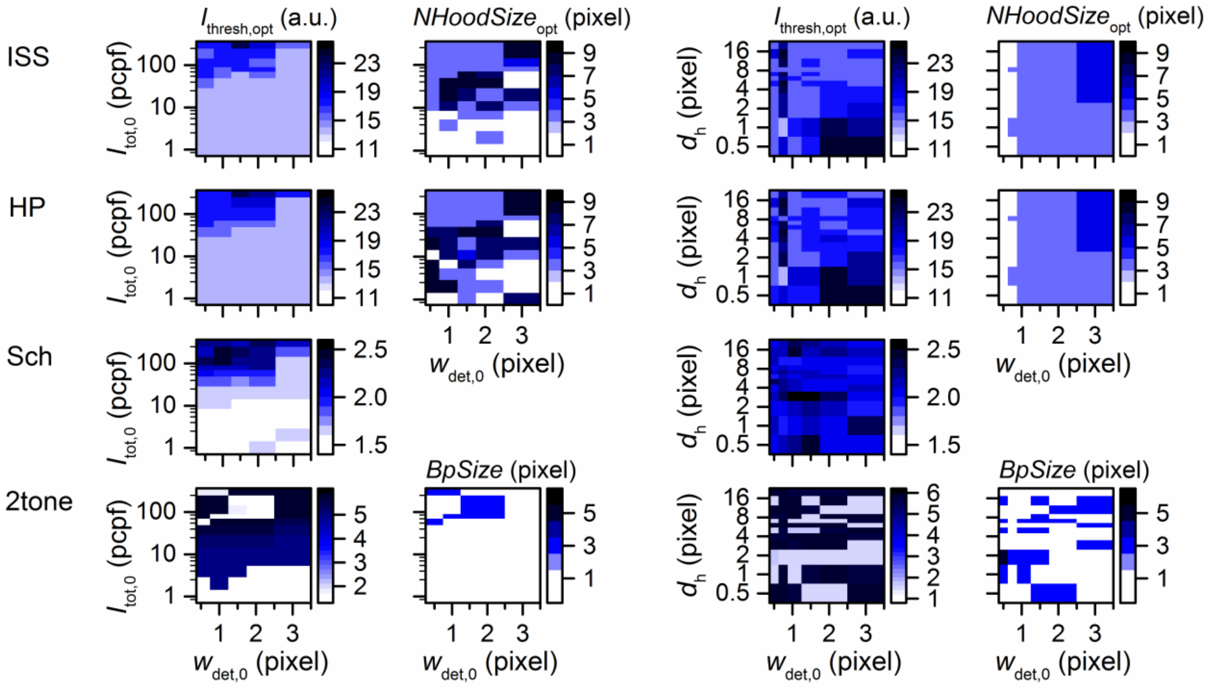


**Fig B.** Method-specific optimization of input parameters for spot detection. Simulated SMVs featuring 12×25 molecules with 1 pc ≤ $I_{tot,0}$ ≤ 300 pc and 0.5 pixel ≤ $w_{det,0}$ ≤ 3 pixel (left), and with intermolecular horizontal distance 0.5 ≤ *IMD* ≤ 16 pixel and 0.5 pixel ≤ $w_{det,0}$ ≤ 3 pixel (right) were generated. For spot detection, the input parameters $I_{thresh}$ (all), *NHoodSize* (ISS and HP), and *BpSize* (2tone) were varied. Heat maps indicate input parameters maximizing spot detection *accuracy*. See also **Figure 5 in main text**.
